# Measurement of charged particle spectra in minimum-bias events from proton–proton collisions at $\sqrt{s}=13\,\text {TeV}$

**DOI:** 10.1140/epjc/s10052-018-6144-y

**Published:** 2018-08-31

**Authors:** A. M. Sirunyan, A. Tumasyan, W. Adam, F. Ambrogi, E. Asilar, T. Bergauer, J. Brandstetter, E. Brondolin, M. Dragicevic, J. Erö, A. Escalante Del Valle, M. Flechl, R. Frühwirth, V. M. Ghete, J. Hrubec, M. Jeitler, N. Krammer, I. Krätschmer, D. Liko, T. Madlener, I. Mikulec, N. Rad, H. Rohringer, J. Schieck, R. Schöfbeck, M. Spanring, D. Spitzbart, A. Taurok, W. Waltenberger, J. Wittmann, C.-E. Wulz, M. Zarucki, V. Chekhovsky, V. Mossolov, J. Suarez Gonzalez, E. A. De Wolf, D. Di Croce, X. Janssen, J. Lauwers, M. Pieters, M. Van De Klundert, H. Van Haevermaet, P. Van Mechelen, N. Van Remortel, S. Abu Zeid, F. Blekman, J. D’Hondt, I. De Bruyn, J. De Clercq, K. Deroover, G. Flouris, D. Lontkovskyi, S. Lowette, I. Marchesini, S. Moortgat, L. Moreels, Q. Python, K. Skovpen, S. Tavernier, W. Van Doninck, P. Van Mulders, I. Van Parijs, D. Beghin, B. Bilin, H. Brun, B. Clerbaux, G. De Lentdecker, H. Delannoy, B. Dorney, G. Fasanella, L. Favart, R. Goldouzian, A. Grebenyuk, A. K. Kalsi, T. Lenzi, J. Luetic, N. Postiau, E. Starling, L. Thomas, C. Vander Velde, P. Vanlaer, D. Vannerom, Q. Wang, T. Cornelis, D. Dobur, A. Fagot, M. Gul, I. Khvastunov, D. Poyraz, C. Roskas, D. Trocino, M. Tytgat, W. Verbeke, B. Vermassen, M. Vit, N. Zaganidis, H. Bakhshiansohi, O. Bondu, S. Brochet, G. Bruno, C. Caputo, P. David, C. Delaere, M. Delcourt, B. Francois, A. Giammanco, G. Krintiras, V. Lemaitre, A. Magitteri, A. Mertens, M. Musich, K. Piotrzkowski, A. Saggio, M. Vidal Marono, S. Wertz, J. Zobec, F. L. Alves, G. A. Alves, L. Brito, G. Correia Silva, C. Hensel, A. Moraes, M. E. Pol, P. Rebello Teles, E. Belchior Batista Das Chagas, W. Carvalho, J. Chinellato, E. Coelho, E. M. Da Costa, G. G. Da Silveira, D. De Jesus Damiao, C. De OliveiraMartins, S. Fonseca De Souza, H. Malbouisson, D. Matos Figueiredo, M. Melo De Almeida, C. Mora Herrera, L. Mundim, H. Nogima, W. L. Prado Da Silva, L. J. Sanchez Rosas, A. Santoro, A. Sznajder, M. Thiel, E. J. Tonelli Manganote, F. Torres Da Silva De Araujo, A. Vilela Pereira, S. Ahuja, C. A. Bernardes, L. Calligaris, T. R. Fernandez Perez Tomei, E. M. Gregores, P. G. Mercadante, S. F. Novaes, Sandra S. Padula, D. Romero Abad, A. Aleksandrov, R. Hadjiiska, P. Iaydjiev, A. Marinov, M. Misheva, M. Rodozov, M. Shopova, G. Sultanov, A. Dimitrov, L. Litov, B. Pavlov, P. Petkov, W. Fang, X. Gao, L. Yuan, M. Ahmad, J. G. Bian, G. M. Chen, H. S. Chen, M. Chen, Y. Chen, C. H. Jiang, D. Leggat, H. Liao, Z. Liu, F. Romeo, S. M. Shaheen, A. Spiezia, J. Tao, C. Wang, Z. Wang, E. Yazgan, H. Zhang, J. Zhao, Y. Ban, G. Chen, J. Li, L. Li, Q. Li, Y. Mao, S. J. Qian, D. Wang, Z. Xu, Y. Wang, C. Avila, A. Cabrera, C. A. Carrillo Montoya, L. F. Chaparro Sierra, C. Florez, C. F. González Hernández, M. A. Segura Delgado, B. Courbon, N. Godinovic, D. Lelas, I. Puljak, T. Sculac, Z. Antunovic, M. Kovac, V. Brigljevic, D. Ferencek, K. Kadija, B. Mesic, A. Starodumov, T. Susa, M. W. Ather, A. Attikis, G. Mavromanolakis, J. Mousa, C. Nicolaou, F. Ptochos, P. A. Razis, H. Rykaczewski, M. Finger, M. Finger, E. Ayala, E. Carrera Jarrin, M. A. Mahmoud, Y. Mohammed, E. Salama, S. Bhowmik, A. Carvalho Antunes De Oliveira, R. K. Dewanjee, K. Ehataht, M. Kadastik, M. Raidal, C. Veelken, P. Eerola, H. Kirschenmann, J. Pekkanen, M. Voutilainen, J. Havukainen, J. K. Heikkilä, T. Järvinen, V. Karimäki, R. Kinnunen, T. Lampén, K. Lassila-Perini, S. Laurila, S. Lehti, T. Lindén, P. Luukka, T. Mäenpää, H. Siikonen, E. Tuominen, J. Tuominiemi, T. Tuuva, M. Besancon, F. Couderc, M. Dejardin, D. Denegri, J. L. Faure, F. Ferri, S. Ganjour, A. Givernaud, P. Gras, G. Hamel de Monchenault, P. Jarry, C. Leloup, E. Locci, J. Malcles, G. Negro, J. Rander, A. Rosowsky, M. Ö. Sahin, M. Titov, A. Abdulsalam, C. Amendola, I. Antropov, F. Beaudette, P. Busson, C. Charlot, R. Granier de Cassagnac, I. Kucher, S. Lisniak, A. Lobanov, J. Martin Blanco, M. Nguyen, C. Ochando, G. Ortona, P. Paganini, P. Pigard, R. Salerno, J. B. Sauvan, Y. Sirois, A. G. Stahl Leiton, A. Zabi, A. Zghiche, J.-L. Agram, J. Andrea, D. Bloch, J.-M. Brom, E. C. Chabert, V. Cherepanov, C. Collard, E. Conte, J.-C. Fontaine, D. Gelé, U. Goerlach, M. Jansová, A.-C. Le Bihan, N. Tonon, P. Van Hove, S. Gadrat, S. Beauceron, C. Bernet, G. Boudoul, N. Chanon, R. Chierici, D. Contardo, P. Depasse, H. El Mamouni, J. Fay, L. Finco, S. Gascon, M. Gouzevitch, G. Grenier, B. Ille, F. Lagarde, I. B. Laktineh, H. Lattaud, M. Lethuillier, L. Mirabito, A. L. Pequegnot, S. Perries, A. Popov, V. Sordini, M. Vander Donckt, S. Viret, S. Zhang, A. Khvedelidze, Z. Tsamalaidze, C. Autermann, L. Feld, M. K. Kiesel, K. Klein, M. Lipinski, M. Preuten, M. P. Rauch, C. Schomakers, J. Schulz, M. Teroerde, B. Wittmer, V. Zhukov, A. Albert, D. Duchardt, M. Endres, M. Erdmann, T. Esch, R. Fischer, S. Ghosh, A. Güth, T. Hebbeker, C. Heidemann, K. Hoepfner, H. Keller, S. Knutzen, L. Mastrolorenzo, M. Merschmeyer, A. Meyer, P. Millet, S. Mukherjee, T. Pook, M. Radziej, H. Reithler, M. Rieger, F. Scheuch, A. Schmidt, D. Teyssier, G. Flügge, O. Hlushchenko, B. Kargoll, T. Kress, A. Künsken, T. Müller, A. Nehrkorn, A. Nowack, C. Pistone, O. Pooth, H. Sert, A. Stahl, M. Aldaya Martin, T. Arndt, C. Asawatangtrakuldee, I. Babounikau, K. Beernaert, O. Behnke, U. Behrens, A. Bermúdez Martínez, D. Bertsche, A. A. Bin Anuar, K. Borras, V. Botta, A. Campbell, P. Connor, C. Contreras-Campana, F. Costanza, V. Danilov, A. De Wit, M. M. Defranchis, C. Diez Pardos, D. Domínguez Damiani, G. Eckerlin, T. Eichhorn, A. Elwood, E. Eren, E. Gallo, A. Geiser, J. M. Grados Luyando, A. Grohsjean, P. Gunnellini, M. Guthoff, A. Harb, J. Hauk, H. Jung, M. Kasemann, J. Keaveney, C. Kleinwort, J. Knolle, D. Krücker, W. Lange, A. Lelek, T. Lenz, K. Lipka, W. Lohmann, R. Mankel, I.-A. Melzer-Pellmann, A. B. Meyer, M. Meyer, M. Missiroli, G. Mittag, J. Mnich, V. Myronenko, S. K. Pflitsch, D. Pitzl, A. Raspereza, M. Savitskyi, P. Saxena, P. Schütze, C. Schwanenberger, R. Shevchenko, A. Singh, N. Stefaniuk, H. Tholen, A. Vagnerini, G. P. Van Onsem, R. Walsh, Y. Wen, K. Wichmann, C. Wissing, O. Zenaiev, R. Aggleton, S. Bein, A. Benecke, V. Blobel, M. Centis Vignali, T. Dreyer, E. Garutti, D. Gonzalez, J. Haller, A. Hinzmann, M. Hoffmann, A. Karavdina, G. Kasieczka, R. Klanner, R. Kogler, N. Kovalchuk, S. Kurz, V. Kutzner, J. Lange, D. Marconi, J. Multhaup, M. Niedziela, D. Nowatschin, A. Perieanu, A. Reimers, O. Rieger, C. Scharf, P. Schleper, S. Schumann, J. Schwandt, J. Sonneveld, H. Stadie, G. Steinbrück, F. M. Stober, M. Stöver, D. Troendle, E. Usai, A. Vanhoefer, B. Vormwald, M. Akbiyik, C. Barth, M. Baselga, S. Baur, E. Butz, R. Caspart, T. Chwalek, F. Colombo, W. De Boer, A. Dierlamm, N. Faltermann, B. Freund, M. Giffels, M. A. Harrendorf, F. Hartmann, S. M. Heindl, U. Husemann, F. Kassel, I. Katkov, S. Kudella, H. Mildner, S. Mitra, M. U. Mozer, Th. Müller, M. Plagge, G. Quast, K. Rabbertz, M. Schröder, I. Shvetsov, G. Sieber, H. J. Simonis, R. Ulrich, S. Wayand, M. Weber, T. Weiler, S. Williamson, C. Wöhrmann, R. Wolf, G. Anagnostou, G. Daskalakis, T. Geralis, A. Kyriakis, D. Loukas, G. Paspalaki, I. Topsis-Giotis, G. Karathanasis, S. Kesisoglou, P. Kontaxakis, A. Panagiotou, N. Saoulidou, E. Tziaferi, K. Vellidis, K. Kousouris, I. Papakrivopoulos, G. Tsipolitis, I. Evangelou, C. Foudas, P. Gianneios, P. Katsoulis, P. Kokkas, S. Mallios, N. Manthos, I. Papadopoulos, E. Paradas, J. Strologas, F. A. Triantis, D. Tsitsonis, M. Csanad, N. Filipovic, P. Major, M. I. Nagy, G. Pasztor, O. Surányi, G. I. Veres, G. Bencze, C. Hajdu, D. Horvath, Á. Hunyadi, F. Sikler, T. Á. Vámi, V. Veszpremi, G. Vesztergombi, N. Beni, S. Czellar, J. Karancsi, A. Makovec, J. Molnar, Z. Szillasi, M. Bartók, P. Raics, Z. L. Trocsanyi, B. Ujvari, S. Choudhury, J. R. Komaragiri, S. Bahinipati, P. Mal, K. Mandal, A. Nayak, D. K. Sahoo, S. K. Swain, S. Bansal, S. B. Beri, V. Bhatnagar, S. Chauhan, R. Chawla, N. Dhingra, R. Gupta, A. Kaur, A. Kaur, M. Kaur, S. Kaur, R. Kumar, P. Kumari, M. Lohan, A. Mehta, K. Sandeep, S. Sharma, J. B. Singh, G. Walia, A. Bhardwaj, B. C. Choudhary, R. B. Garg, M. Gola, S. Keshri, Ashok Kumar, S. Malhotra, M. Naimuddin, P. Priyanka, K. Ranjan, Aashaq Shah, R. Sharma, R. Bhardwaj, M. Bharti, R. Bhattacharya, S. Bhattacharya, U. Bhawandeep, D. Bhowmik, S. Dey, S. Dutt, S. Dutta, S. Ghosh, K. Mondal, S. Nandan, A. Purohit, P. K. Rout, A. Roy, S. Roy Chowdhury, S. Sarkar, M. Sharan, B. Singh, S. Thakur, P. K. Behera, R. Chudasama, D. Dutta, V. Jha, V. Kumar, P. K. Netrakanti, L. M. Pant, P. Shukla, T. Aziz, M. A. Bhat, S. Dugad, B. Mahakud, G. B. Mohanty, N. Sur, B. Sutar, RavindraKumar Verma, S. Banerjee, S. Bhattacharya, S. Chatterjee, P. Das, M. Guchait, Sa. Jain, S. Kumar, M. Maity, G. Majumder, K. Mazumdar, N. Sahoo, T. Sarkar, S. Chauhan, S. Dube, V. Hegde, A. Kapoor, K. Kothekar, S. Pandey, A. Rane, S. Sharma, S. Chenarani, E. Eskandari Tadavani, S. M. Etesami, M. Khakzad, M. Mohammadi Najafabadi, M. Naseri, F. Rezaei Hosseinabadi, B. Safarzadeh, M. Zeinali, M. Felcini, M. Grunewald, M. Abbrescia, C. Calabria, A. Colaleo, D. Creanza, L. Cristella, N. De Filippis, M. De Palma, A. Di Florio, F. Errico, L. Fiore, A. Gelmi, G. Iaselli, S. Lezki, G. Maggi, M. Maggi, G. Miniello, S. My, S. Nuzzo, A. Pompili, G. Pugliese, R. Radogna, A. Ranieri, G. Selvaggi, A. Sharma, L. Silvestris, R. Venditti, P. Verwilligen, G. Zito, G. Abbiendi, C. Battilana, D. Bonacorsi, L. Borgonovi, S. Braibant-Giacomelli, L. Brigliadori, R. Campanini, P. Capiluppi, A. Castro, F. R. Cavallo, S. S. Chhibra, G. Codispoti, M. Cuffiani, G. M. Dallavalle, F. Fabbri, A. Fanfani, P. Giacomelli, C. Grandi, L. Guiducci, S. Marcellini, G. Masetti, A. Montanari, F. L. Navarria, A. Perrotta, A. M. Rossi, T. Rovelli, G. P. Siroli, N. Tosi, S. Albergo, A. Di Mattia, R. Potenza, A. Tricomi, C. Tuve, G. Barbagli, K. Chatterjee, V. Ciulli, C. Civinini, R. D’Alessandro, E. Focardi, G. Latino, P. Lenzi, M. Meschini, S. Paoletti, L. Russo, G. Sguazzoni, D. Strom, L. Viliani, L. Benussi, S. Bianco, F. Fabbri, D. Piccolo, F. Primavera, F. Ferro, F. Ravera, E. Robutti, S. Tosi, A. Benaglia, A. Beschi, L. Brianza, F. Brivio, V. Ciriolo, S. Di Guida, M. E. Dinardo, S. Fiorendi, S. Gennai, A. Ghezzi, P. Govoni, M. Malberti, S. Malvezzi, R. A. Manzoni, A. Massironi, D. Menasce, L. Moroni, M. Paganoni, D. Pedrini, S. Ragazzi, T. Tabarelli de Fatis, S. Buontempo, N. Cavallo, A. Di Crescenzo, F. Fabozzi, F. Fienga, G. Galati, A. O. M. Iorio, W. A. Khan, L. Lista, S. Meola, P. Paolucci, C. Sciacca, E. Voevodina, P. Azzi, N. Bacchetta, L. Benato, A. Boletti, A. Bragagnolo, R. Carlin, P. Checchia, M. Dall’Osso, P. De Castro Manzano, T. Dorigo, F. Gasparini, U. Gasparini, A. Gozzelino, S. Lacaprara, P. Lujan, M. Margoni, A. T. Meneguzzo, N. Pozzobon, P. Ronchese, R. Rossin, F. Simonetto, A. Tiko, E. Torassa, S. Ventura, M. Zanetti, P. Zotto, G. Zumerle, A. Braghieri, A. Magnani, P. Montagna, S. P. Ratti, V. Re, M. Ressegotti, C. Riccardi, P. Salvini, I. Vai, P. Vitulo, L. Alunni Solestizi, M. Biasini, G. M. Bilei, C. Cecchi, D. Ciangottini, L. Fanò, P. Lariccia, E. Manoni, G. Mantovani, V. Mariani, M. Menichelli, A. Rossi, A. Santocchia, D. Spiga, K. Androsov, P. Azzurri, G. Bagliesi, L. Bianchini, T. Boccali, L. Borrello, R. Castaldi, M. A. Ciocci, R. Dell’Orso, G. Fedi, L. Giannini, A. Giassi, M. T. Grippo, F. Ligabue, E. Manca, G. Mandorli, A. Messineo, F. Palla, A. Rizzi, P. Spagnolo, R. Tenchini, G. Tonelli, A. Venturi, P. G. Verdini, L. Barone, F. Cavallari, M. Cipriani, N. Daci, D. Del Re, E. Di Marco, M. Diemoz, S. Gelli, E. Longo, B. Marzocchi, P. Meridiani, G. Organtini, F. Pandolfi, R. Paramatti, F. Preiato, S. Rahatlou, C. Rovelli, F. Santanastasio, N. Amapane, R. Arcidiacono, S. Argiro, M. Arneodo, N. Bartosik, R. Bellan, C. Biino, N. Cartiglia, F. Cenna, M. Costa, R. Covarelli, N. Demaria, B. Kiani, C. Mariotti, S. Maselli, E. Migliore, V. Monaco, E. Monteil, M. Monteno, M. M. Obertino, L. Pacher, N. Pastrone, M. Pelliccioni, G. L. Pinna Angioni, A. Romero, M. Ruspa, R. Sacchi, K. Shchelina, V. Sola, A. Solano, A. Staiano, S. Belforte, V. Candelise, M. Casarsa, F. Cossutti, G. Della Ricca, F. Vazzoler, A. Zanetti, D. H. Kim, G. N. Kim, M. S. Kim, J. Lee, S. Lee, S. W. Lee, C. S. Moon, Y. D. Oh, S. Sekmen, D. C. Son, Y. C. Yang, H. Kim, D. H. Moon, G. Oh, J. Goh, T. J. Kim, S. Cho, S. Choi, Y. Go, D. Gyun, S. Ha, B. Hong, Y. Jo, K. Lee, K. S. Lee, S. Lee, J. Lim, S. K. Park, Y. Roh, H. S. Kim, J. Almond, J. Kim, J. S. Kim, H. Lee, K. Lee, K. Nam, S. B. Oh, B. C. Radburn-Smith, S. H. Seo, U. K. Yang, H. D. Yoo, G. B. Yu, H. Kim, J. H. Kim, J. S. H. Lee, I. C. Park, Y. Choi, C. Hwang, J. Lee, I. Yu, V. Dudenas, A. Juodagalvis, J. Vaitkus, I. Ahmed, Z. A. Ibrahim, M. A. B. Md Ali, F. Mohamad Idris, W. A. T. Wan Abdullah, M. N. Yusli, Z. Zolkapli, H. Castilla-Valdez, E. De La Cruz-Burelo, I. Heredia-De La Cruz, R. Lopez-Fernandez, J. Mejia Guisao, R. I. Rabadan-Trejo, G. Ramirez-Sanchez, R Reyes-Almanza, A. Sanchez-Hernandez, S. Carrillo Moreno, C. Oropeza Barrera, F. Vazquez Valencia, J. Eysermans, I. Pedraza, H. A. Salazar Ibarguen, C. Uribe Estrada, A. Morelos Pineda, D. Krofcheck, S. Bheesette, P. H. Butler, A. Ahmad, M. Ahmad, M. I. Asghar, Q. Hassan, H. R. Hoorani, A. Saddique, M. A. Shah, M. Shoaib, M. Waqas, H. Bialkowska, M. Bluj, B. Boimska, T. Frueboes, M. Górski, M. Kazana, K. Nawrocki, M. Szleper, P. Traczyk, P. Zalewski, K. Bunkowski, A. Byszuk, K. Doroba, A. Kalinowski, M. Konecki, J. Krolikowski, M. Misiura, M. Olszewski, A. Pyskir, M. Walczak, P. Bargassa, C. Beirão Da Cruz E Silva, A. Di Francesco, P. Faccioli, B. Galinhas, M. Gallinaro, J. Hollar, N. Leonardo, L. Lloret Iglesias, M. V. Nemallapudi, J. Seixas, G. Strong, O. Toldaiev, D. Vadruccio, J. Varela, A. Baginyan, I. Golutvin, V. Karjavin, I. Kashunin, V. Korenkov, G. Kozlov, A. Lanev, A. Malakhov, V. Matveev, P. Moisenz, V. Palichik, V. Perelygin, S. Shmatov, N. Skatchkov, V. Smirnov, V. Trofimov, B. S. Yuldashev, A. Zarubin, V. Zhiltsov, V. Golovtsov, Y. Ivanov, V. Kim, E. Kuznetsova, P. Levchenko, V. Murzin, V. Oreshkin, I. Smirnov, D. Sosnov, V. Sulimov, L. Uvarov, S. Vavilov, A. Vorobyev, Yu. Andreev, A. Dermenev, S. Gninenko, N. Golubev, A. Karneyeu, M. Kirsanov, N. Krasnikov, A. Pashenkov, D. Tlisov, A. Toropin, V. Epshteyn, V. Gavrilov, N. Lychkovskaya, V. Popov, I. Pozdnyakov, G. Safronov, A. Spiridonov, A. Stepennov, V. Stolin, M. Toms, E. Vlasov, A. Zhokin, T. Aushev, A. Bylinkin, R Chistov, P. Parygin, D. Philippov, S. Polikarpov, E. Popova, E. Tarkovskii, E. Zhemchugov, V. Andreev, M. Azarkin, I. Dremin, M. Kirakosyan, S. V. Rusakov, A. Terkulov, A. Baskakov, A. Belyaev, E. Boos, A. Ershov, A. Gribushin, L. Khein, V. Klyukhin, O. Kodolova, I. Lokhtin, O. Lukina, I. Miagkov, S. Obraztsov, S. Petrushanko, V. Savrin, A. Snigirev, V. Blinov, T. Dimova, L. Kardapoltsev, D. Shtol, Y. Skovpen, I. Azhgirey, I. Bayshev, S. Bitioukov, D. Elumakhov, A. Godizov, V. Kachanov, A. Kalinin, D. Konstantinov, P. Mandrik, V. Petrov, R. Ryutin, S. Slabospitskii, A. Sobol, S. Troshin, N. Tyurin, A. Uzunian, A. Volkov, A. Babaev, P. Adzic, P. Cirkovic, D. Devetak, M. Dordevic, J. Milosevic, J. Alcaraz Maestre, A. Álvarez Fernández, I. Bachiller, M. Barrio Luna, J. A. Brochero Cifuentes, M. Cerrada, N. Colino, B. De La Cruz, A. Delgado Peris, C. Fernandez Bedoya, J. P. Fernández Ramos, J. Flix, M. C. Fouz, O. Gonzalez Lopez, S. Goy Lopez, J. M. Hernandez, M. I. Josa, D. Moran, A. Pérez-Calero Yzquierdo, J. Puerta Pelayo, I. Redondo, L. Romero, M. S. Soares, A. Triossi, C. Albajar, J. F. de Trocóniz, J. Cuevas, C. Erice, J. Fernandez Menendez, S. Folgueras, I. Gonzalez Caballero, J. R. González Fernández, E. Palencia Cortezon, V. Rodríguez Bouza, S. Sanchez Cruz, P. Vischia, J. M. Vizan Garcia, I. J. Cabrillo, A. Calderon, B. Chazin Quero, J. Duarte Campderros, M. Fernandez, P. J. Fernández Manteca, A. García Alonso, J. Garcia-Ferrero, G. Gomez, A. Lopez Virto, J. Marco, C. Martinez Rivero, P. Martinez Ruiz del Arbol, F. Matorras, J. Piedra Gomez, C. Prieels, T. Rodrigo, A. Ruiz-Jimeno, L. Scodellaro, N. Trevisani, I. Vila, R. Vilar Cortabitarte, D. Abbaneo, B. Akgun, E. Auffray, P. Baillon, A. H. Ball, D. Barney, J. Bendavid, M. Bianco, A. Bocci, C. Botta, T. Camporesi, M. Cepeda, G. Cerminara, E. Chapon, Y. Chen, G. Cucciati, D. d’Enterria, A. Dabrowski, V. Daponte, A. David, A. De Roeck, N. Deelen, M. Dobson, T. du Pree, M. Dünser, N. Dupont, A. Elliott-Peisert, P. Everaerts, F. Fallavollita, D. Fasanella, G. Franzoni, J. Fulcher, W. Funk, D. Gigi, A. Gilbert, K. Gill, F. Glege, D. Gulhan, J. Hegeman, V. Innocente, A. Jafari, P. Janot, O. Karacheban, J. Kieseler, V. Knünz, A. Kornmayer, M. Krammer, C. Lange, P. Lecoq, C. Lourenço, L. Malgeri, M. Mannelli, F. Meijers, J. A. Merlin, S. Mersi, E. Meschi, P. Milenovic, F. Moortgat, M. Mulders, H. Neugebauer, J. Ngadiuba, S. Orfanelli, L. Orsini, F. Pantaleo, L. Pape, E. Perez, M. Peruzzi, A. Petrilli, G. Petrucciani, A. Pfeiffer, M. Pierini, F. M. Pitters, D. Rabady, A. Racz, T. Reis, G. Rolandi, M. Rovere, H. Sakulin, C. Schäfer, C. Schwick, M. Seidel, M. Selvaggi, A. Sharma, P. Silva, P. Sphicas, A. Stakia, J. Steggemann, M. Tosi, D. Treille, A. Tsirou, V. Veckalns, M. Verweij, W. D. Zeuner, W. Bertl, L. Caminada, K. Deiters, W. Erdmann, R. Horisberger, Q. Ingram, H. C. Kaestli, D. Kotlinski, U. Langenegger, T. Rohe, S. A. Wiederkehr, M. Backhaus, L. Bäni, P. Berger, N. Chernyavskaya, G. Dissertori, M. Dittmar, M. Donegà, C. Dorfer, C. Grab, C. Heidegger, D. Hits, J. Hoss, T. Klijnsma, W. Lustermann, M. Marionneau, M. T. Meinhard, D. Meister, F. Micheli, P. Musella, F. Nessi-Tedaldi, J. Pata, F. Pauss, G. Perrin, L. Perrozzi, S. Pigazzini, M. Quittnat, M. Reichmann, D. Ruini, D. A. Sanz Becerra, M. Schönenberger, L. Shchutska, V. R. Tavolaro, K. Theofilatos, M. L. Vesterbacka Olsson, R. Wallny, D. H. Zhu, T. K. Aarrestad, C. Amsler, D. Brzhechko, M. F. Canelli, A. De Cosa, R. Del Burgo, S. Donato, C. Galloni, T. Hreus, B. Kilminster, I. Neutelings, D. Pinna, G. Rauco, P. Robmann, D. Salerno, K. Schweiger, C. Seitz, Y. Takahashi, A. Zucchetta, Y. H. Chang, K. y. Cheng, T. H. Doan, Sh. Jain, R. Khurana, C. M. Kuo, W. Lin, A. Pozdnyakov, S. S. Yu, P. Chang, Y. Chao, K. F. Chen, P. H. Chen, W.-S. Hou, Arun Kumar, Y. Y. Li, R.-S. Lu, E. Paganis, A. Psallidas, A. Steen, J. f. Tsai, B. Asavapibhop, N. Srimanobhas, N. Suwonjandee, A. Bat, F. Boran, S. Cerci, S. Damarseckin, Z. S. Demiroglu, C. Dozen, E. Eskut, S. Girgis, G. Gokbulut, Y. Guler, E. Gurpinar, I. Hos, E. E. Kangal, O. Kara, U. Kiminsu, M. Oglakci, G. Onengut, K. Ozdemir, S. Ozturk, D. Sunar Cerci, B. Tali, U. G. Tok, H. Topakli, S. Turkcapar, I. S. Zorbakir, C. Zorbilmez, B. Isildak, G. Karapinar, M. Yalvac, M. Zeyrek, I. O. Atakisi, E. Gülmez, M. Kaya, O. Kaya, S. Tekten, E. A. Yetkin, M. N. Agaras, S. Atay, A. Cakir, K. Cankocak, Y. Komurcu, S. Sen, B. Grynyov, L. Levchuk, T. Alexander, F. Ball, L. Beck, J. J. Brooke, D. Burns, E. Clement, D. Cussans, O. Davignon, H. Flacher, J. Goldstein, G. P. Heath, H. F. Heath, L. Kreczko, D. M. Newbold, S. Paramesvaran, B. Penning, T. Sakuma, D. Smith, V. J. Smith, J. Taylor, K. W. Bell, A. Belyaev, C. Brew, R. M. Brown, D. Cieri, D. J. A. Cockerill, J. A. Coughlan, K. Harder, S. Harper, J. Linacre, E. Olaiya, D. Petyt, C. H. Shepherd-Themistocleous, A. Thea, I. R. Tomalin, T. Williams, W. J. Womersley, G. Auzinger, R. Bainbridge, P. Bloch, J. Borg, S. Breeze, O. Buchmuller, A. Bundock, S. Casasso, D. Colling, L. Corpe, P. Dauncey, G. Davies, M. Della Negra, R. Di Maria, Y. Haddad, G. Hall, G. Iles, T. James, M. Komm, C. Laner, L. Lyons, A.-M. Magnan, S. Malik, A. Martelli, J. Nash, A. Nikitenko, V. Palladino, M. Pesaresi, A. Richards, A. Rose, E. Scott, C. Seez, A. Shtipliyski, G. Singh, M. Stoye, T. Strebler, S. Summers, A. Tapper, K. Uchida, T. Virdee, N. Wardle, D. Winterbottom, J. Wright, S. C. Zenz, J. E. Cole, P. R. Hobson, A. Khan, P. Kyberd, C. K. Mackay, A. Morton, I. D. Reid, L. Teodorescu, S. Zahid, K. Call, J. Dittmann, K. Hatakeyama, H. Liu, C. Madrid, B. Mcmaster, N. Pastika, C. Smith, R. Bartek, A. Dominguez, A. Buccilli, S. I. Cooper, C. Henderson, P. Rumerio, C. West, D. Arcaro, T. Bose, D. Gastler, D. Rankin, C. Richardson, J. Rohlf, L. Sulak, D. Zou, G. Benelli, X. Coubez, D. Cutts, M. Hadley, J. Hakala, U. Heintz, J. M. Hogan, K. H. M. Kwok, E. Laird, G. Landsberg, J. Lee, Z. Mao, M. Narain, J. Pazzini, S. Piperov, S. Sagir, R. Syarif, D. Yu, R. Band, C. Brainerd, R. Breedon, D. Burns, M. Calderon De LaBarca Sanchez, M. Chertok, J. Conway, R. Conway, P. T. Cox, R. Erbacher, C. Flores, G. Funk, W. Ko, O. Kukral, R. Lander, C. Mclean, M. Mulhearn, D. Pellett, J. Pilot, S. Shalhout, M. Shi, D. Stolp, D. Taylor, K. Tos, M. Tripathi, Z. Wang, F. Zhang, M. Bachtis, C. Bravo, R. Cousins, A. Dasgupta, A. Florent, J. Hauser, M. Ignatenko, N. Mccoll, S. Regnard, D. Saltzberg, C. Schnaible, V. Valuev, E. Bouvier, K. Burt, R. Clare, J. W. Gary, S. M. A. Ghiasi Shirazi, G. Hanson, G. Karapostoli, E. Kennedy, F. Lacroix, O. R. Long, M. Olmedo Negrete, M. I. Paneva, W. Si, L. Wang, H. Wei, S. Wimpenny, B. R. Yates, J. G. Branson, S. Cittolin, M. Derdzinski, R. Gerosa, D. Gilbert, B. Hashemi, A. Holzner, D. Klein, G. Kole, V. Krutelyov, J. Letts, M. Masciovecchio, D. Olivito, S. Padhi, M. Pieri, M. Sani, V. Sharma, S. Simon, M. Tadel, A. Vartak, S. Wasserbaech, J. Wood, F. Würthwein, A. Yagil, G. Zevi Della Porta, N. Amin, R. Bhandari, J. Bradmiller-Feld, C. Campagnari, M. Citron, A. Dishaw, V. Dutta, M. Franco Sevilla, L. Gouskos, R. Heller, J. Incandela, A. Ovcharova, H. Qu, J. Richman, D. Stuart, I. Suarez, S. Wang, J. Yoo, D. Anderson, A. Bornheim, J. Bunn, J. M. Lawhorn, H. B. Newman, T. Q. Nguyen, M. Spiropulu, J. R. Vlimant, R. Wilkinson, S. Xie, Z. Zhang, R. Y. Zhu, M. B. Andrews, T. Ferguson, T. Mudholkar, M. Paulini, M. Sun, I. Vorobiev, M. Weinberg, J. P. Cumalat, W. T. Ford, F. Jensen, A. Johnson, M. Krohn, S. Leontsinis, E. MacDonald, T. Mulholland, K. Stenson, K. A. Ulmer, S. R. Wagner, J. Alexander, J. Chaves, Y. Cheng, J. Chu, A. Datta, K. Mcdermott, N. Mirman, J. R. Patterson, D. Quach, A. Rinkevicius, A. Ryd, L. Skinnari, L. Soffi, S. M. Tan, Z. Tao, J. Thom, J. Tucker, P. Wittich, M. Zientek, S. Abdullin, M. Albrow, M. Alyari, G. Apollinari, A. Apresyan, A. Apyan, S. Banerjee, L. A. T. Bauerdick, A. Beretvas, J. Berryhill, P. C. Bhat, G. Bolla, K. Burkett, J. N. Butler, A. Canepa, G. B. Cerati, H. W. K. Cheung, F. Chlebana, M. Cremonesi, J. Duarte, V. D. Elvira, J. Freeman, Z. Gecse, E. Gottschalk, L. Gray, D. Green, S. Grünendahl, O. Gutsche, J. Hanlon, R. M. Harris, S. Hasegawa, J. Hirschauer, Z. Hu, B. Jayatilaka, S. Jindariani, M. Johnson, U. Joshi, B. Klima, M. J. Kortelainen, B. Kreis, S. Lammel, D. Lincoln, R. Lipton, M. Liu, T. Liu, J. Lykken, K. Maeshima, J. M. Marraffino, D. Mason, P. McBride, P. Merkel, S. Mrenna, S. Nahn, V. O’Dell, K. Pedro, C. Pena, O. Prokofyev, G. Rakness, L. Ristori, A. Savoy-Navarro, B. Schneider, E. Sexton-Kennedy, A. Soha, W. J. Spalding, L. Spiegel, S. Stoynev, J. Strait, N. Strobbe, L. Taylor, S. Tkaczyk, N. V. Tran, L. Uplegger, E. W. Vaandering, C. Vernieri, M. Verzocchi, R. Vidal, M. Wang, H. A. Weber, A. Whitbeck, D. Acosta, P. Avery, P. Bortignon, D. Bourilkov, A. Brinkerhoff, L. Cadamuro, A. Carnes, M. Carver, D. Curry, R. D. Field, S. V. Gleyzer, B. M. Joshi, J. Konigsberg, A. Korytov, P. Ma, K. Matchev, H. Mei, G. Mitselmakher, K. Shi, D. Sperka, J. Wang, S. Wang, Y. R. Joshi, S. Linn, A. Ackert, T. Adams, A. Askew, S. Hagopian, V. Hagopian, K. F. Johnson, T. Kolberg, G. Martinez, T. Perry, H. Prosper, A. Saha, A. Santra, V. Sharma, R. Yohay, M. M. Baarmand, V. Bhopatkar, S. Colafranceschi, M. Hohlmann, D. Noonan, M. Rahmani, T. Roy, F. Yumiceva, M. R. Adams, L. Apanasevich, D. Berry, R. R. Betts, R. Cavanaugh, X. Chen, S. Dittmer, O. Evdokimov, C. E. Gerber, D. A. Hangal, D. J. Hofman, K. Jung, J. Kamin, C. Mills, I. D. Sandoval Gonzalez, M. B. Tonjes, N. Varelas, H. Wang, Z. Wu, J. Zhang, M. Alhusseini, B. Bilki, W. Clarida, K. Dilsiz, S. Durgut, R. P. Gandrajula, M. Haytmyradov, V. Khristenko, J.-P. Merlo, A. Mestvirishvili, A. Moeller, J. Nachtman, H. Ogul, Y. Onel, F. Ozok, A. Penzo, C. Snyder, E. Tiras, J. Wetzel, B. Blumenfeld, A. Cocoros, N. Eminizer, D. Fehling, L. Feng, A. V. Gritsan, W. T. Hung, P. Maksimovic, J. Roskes, U. Sarica, M. Swartz, M. Xiao, C. You, A. Al-bataineh, P. Baringer, A. Bean, S. Boren, J. Bowen, J. Castle, S. Khalil, A. Kropivnitskaya, D. Majumder, W. Mcbrayer, M. Murray, C. Rogan, S. Sanders, E. Schmitz, J. D. Tapia Takaki, Q. Wang, A. Ivanov, K. Kaadze, D. Kim, Y. Maravin, D. R. Mendis, T. Mitchell, A. Modak, A. Mohammadi, L. K. Saini, N. Skhirtladze, F. Rebassoo, D. Wright, A. Baden, O. Baron, A. Belloni, S. C. Eno, Y. Feng, C. Ferraioli, N. J. Hadley, S. Jabeen, G. Y. Jeng, R. G. Kellogg, J. Kunkle, A. C. Mignerey, F. Ricci-Tam, Y. H. Shin, A. Skuja, S. C. Tonwar, K. Wong, D. Abercrombie, B. Allen, V. Azzolini, R. Barbieri, A. Baty, G. Bauer, R. Bi, S. Brandt, W. Busza, I. A. Cali, M. D’Alfonso, Z. Demiragli, G. Gomez Ceballos, M. Goncharov, P. Harris, D. Hsu, M. Hu, Y. Iiyama, G. M. Innocenti, M. Klute, D. Kovalskyi, Y.-J. Lee, A. Levin, P. D. Luckey, B. Maier, A. C. Marini, C. Mcginn, C. Mironov, S. Narayanan, X. Niu, C. Paus, C. Roland, G. Roland, G. S. F. Stephans, K. Sumorok, K. Tatar, D. Velicanu, J. Wang, T. W. Wang, B. Wyslouch, S. Zhaozhong, A. C. Benvenuti, R. M. Chatterjee, A. Evans, P. Hansen, S. Kalafut, Y. Kubota, Z. Lesko, J. Mans, S. Nourbakhsh, N. Ruckstuhl, R. Rusack, J. Turkewitz, M. A. Wadud, J. G. Acosta, S. Oliveros, E. Avdeeva, K. Bloom, D. R. Claes, C. Fangmeier, F. Golf, R. Gonzalez Suarez, R. Kamalieddin, I. Kravchenko, J. Monroy, J. E. Siado, G. R. Snow, B. Stieger, A. Godshalk, C. Harrington, I. Iashvili, A. Kharchilava, D. Nguyen, A. Parker, S. Rappoccio, B. Roozbahani, G. Alverson, E. Barberis, C. Freer, A. Hortiangtham, D. M. Morse, T. Orimoto, R. Teixeira De Lima, T. Wamorkar, B. Wang, A. Wisecarver, D. Wood, S. Bhattacharya, O. Charaf, K. A. Hahn, N. Mucia, N. Odell, M. H. Schmitt, K. Sung, M. Trovato, M. Velasco, R. Bucci, N. Dev, M. Hildreth, K. Hurtado Anampa, C. Jessop, D. J. Karmgard, N. Kellams, K. Lannon, W. Li, N. Loukas, N. Marinelli, F. Meng, C. Mueller, Y. Musienko, M. Planer, A. Reinsvold, R. Ruchti, P. Siddireddy, G. Smith, S. Taroni, M. Wayne, A. Wightman, M. Wolf, A. Woodard, J. Alimena, L. Antonelli, B. Bylsma, L. S. Durkin, S. Flowers, B. Francis, A. Hart, C. Hill, W. Ji, T. Y. Ling, W. Luo, B. L. Winer, H. W. Wulsin, S. Cooperstein, P. Elmer, J. Hardenbrook, P. Hebda, S. Higginbotham, A. Kalogeropoulos, D. Lange, M. T. Lucchini, J. Luo, D. Marlow, K. Mei, I. Ojalvo, J. Olsen, C. Palmer, P. Piroué, J. Salfeld-Nebgen, D. Stickland, C. Tully, S. Malik, S. Norberg, A. Barker, V. E. Barnes, S. Das, L. Gutay, M. Jones, A. W. Jung, A. Khatiwada, D. H. Miller, N. Neumeister, C. C. Peng, H. Qiu, J. F. Schulte, J. Sun, F. Wang, R. Xiao, W. Xie, T. Cheng, J. Dolen, N. Parashar, Z. Chen, K. M. Ecklund, S. Freed, F. J. M. Geurts, M. Guilbaud, M. Kilpatrick, W. Li, B. Michlin, B. P. Padley, J. Roberts, J. Rorie, W. Shi, Z. Tu, J. Zabel, A. Zhang, A. Bodek, P. de Barbaro, R. Demina, Y. t. Duh, J. L. Dulemba, C. Fallon, T. Ferbel, M. Galanti, A. Garcia-Bellido, J. Han, O. Hindrichs, A. Khukhunaishvili, K. H. Lo, P. Tan, R. Taus, M. Verzetti, A. Agapitos, J. P. Chou, Y. Gershtein, T. A. Gómez Espinosa, E. Halkiadakis, M. Heindl, E. Hughes, S. Kaplan, R. Kunnawalkam Elayavalli, S. Kyriacou, A. Lath, R. Montalvo, K. Nash, M. Osherson, H. Saka, S. Salur, S. Schnetzer, D. Sheffield, S. Somalwar, R. Stone, S. Thomas, P. Thomassen, M. Walker, A. G. Delannoy, J. Heideman, G. Riley, K. Rose, S. Spanier, K. Thapa, O. Bouhali, A. Castaneda Hernandez, A. Celik, M. Dalchenko, M. De Mattia, A. Delgado, S. Dildick, R. Eusebi, J. Gilmore, T. Huang, T. Kamon, S. Luo, R. Mueller, Y. Pakhotin, R. Patel, A. Perloff, L. Perniè, D. Rathjens, A. Safonov, A. Tatarinov, N. Akchurin, J. Damgov, F. De Guio, P. R. Dudero, S. Kunori, K. Lamichhane, S. W. Lee, T. Mengke, S. Muthumuni, T. Peltola, S. Undleeb, I. Volobouev, Z. Wang, S. Greene, A. Gurrola, R. Janjam, W. Johns, C. Maguire, A. Melo, H. Ni, K. Padeken, J. D. Ruiz Alvarez, P. Sheldon, S. Tuo, J. Velkovska, Q. Xu, M. W. Arenton, P. Barria, B. Cox, R. Hirosky, M. Joyce, A. Ledovskoy, H. Li, C. Neu, T. Sinthuprasith, Y. Wang, E. Wolfe, F. Xia, R. Harr, P. E. Karchin, N. Poudyal, J. Sturdy, P. Thapa, S. Zaleski, M. Brodski, J. Buchanan, C. Caillol, D. Carlsmith, S. Dasu, L. Dodd, S. Duric, B. Gomber, M. Grothe, M. Herndon, A. Hervé, U. Hussain, P. Klabbers, A. Lanaro, A. Levine, K. Long, R. Loveless, T. Ruggles, A. Savin, N. Smith, W. H. Smith, N. Woods, CMS Collaboration

**Affiliations:** 1 Yerevan Physics Institute, Yerevan, Armenia; 2 Institut für Hochenergiephysik, Wien, Austria; 3 Institute for Nuclear Problems, Minsk, Belarus; 4 Universiteit Antwerpen, Antwerpen, Belgium; 5 Vrije Universiteit Brussel, Brussel, Belgium; 6 Université Libre de Bruxelles, Brussels, Belgium; 7 Ghent University, Ghent, Belgium; 8 Université Catholique de Louvain, Louvain-la-Neuve, Belgium; 9 Centro Brasileiro de Pesquisas Fisicas, Rio de Janeiro, Brazil; 10 Universidade do Estado do Rio de Janeiro, Rio de Janeiro, Brazil; 11 Universidade Estadual Paulista, Universidade Federal do ABC, São Paulo, Brazil; 12 Institute for Nuclear Research and Nuclear Energy, Bulgarian Academy of Sciences, Sofia, Bulgaria; 13 University of Sofia, Sofia, Bulgaria; 14 Beihang University, Beijing, China; 15 Institute of High Energy Physics, Beijing, China; 16 State Key Laboratory of Nuclear Physics and Technology, Peking University, Beijing, China; 17 Tsinghua University, Beijing, China; 18 Universidad de Los Andes, Bogotá, Colombia; 19 University of Split, Faculty of Electrical Engineering, Mechanical Engineering and Naval Architecture, Split, Croatia; 20 University of Split, Faculty of Science, Split, Croatia; 21 Institute Rudjer Boskovic, Zagreb, Croatia; 22 University of Cyprus, Nicosia, Cyprus; 23 Charles University, Prague, Czech Republic; 24 Escuela Politecnica Nacional, Quito, Ecuador; 25 Universidad San Francisco de Quito, Quito, Ecuador; 26 Academy of Scientific Research and Technology of the Arab Republic of Egypt, Egyptian Network of High Energy Physics, Cairo, Egypt; 27 National Institute of Chemical Physics and Biophysics, Tallinn, Estonia; 28 Department of Physics, University of Helsinki, Helsinki, Finland; 29 Helsinki Institute of Physics, Helsinki, Finland; 30 Lappeenranta University of Technology, Lappeenranta, Finland; 31 IRFU, CEA, Université Paris-Saclay, Gif-sur-Yvette, France; 32 Laboratoire Leprince-Ringuet, Ecole polytechnique, CNRS/IN2P3, Université Paris-Saclay, Palaiseau, France; 33 Université de Strasbourg, CNRS, IPHC UMR 7178, 67000 Strasbourg, France; 34 Centre de Calcul de l’Institut National de Physique Nucleaire et de Physique des Particules, CNRS/IN2P3, Villeurbanne, France; 35 Université de Lyon, Université Claude Bernard Lyon 1, CNRS-IN2P3, Institut de Physique Nucléaire de Lyon, Villeurbanne, France; 36 Georgian Technical University, Tbilisi, Georgia; 37 Tbilisi State University, Tbilisi, Georgia; 38 RWTH Aachen University, I. Physikalisches Institut, Aachen, Germany; 39 RWTH Aachen University, III. Physikalisches Institut A, Aachen, Germany; 40 RWTH Aachen University, III. Physikalisches Institut B, Aachen, Germany; 41 Deutsches Elektronen-Synchrotron, Hamburg, Germany; 42 University of Hamburg, Hamburg, Germany; 43 Karlsruher Institut fuer Technology, Karlsruhe, Germany; 44 Institute of Nuclear and Particle Physics (INPP), NCSR Demokritos, Aghia Paraskevi, Greece; 45 National and Kapodistrian University of Athens, Athens, Greece; 46 National Technical University of Athens, Athens, Greece; 47 University of Ioánnina, Ioannina, Greece; 48 MTA-ELTE Lendület CMS Particle and Nuclear Physics Group, Eötvös Loránd University, Budapest, Hungary; 49 Wigner Research Centre for Physics, Budapest, Hungary; 50 Institute of Nuclear Research ATOMKI, Debrecen, Hungary; 51 Institute of Physics, University of Debrecen, Debrecen, Hungary; 52 Indian Institute of Science (IISc), Bangalore, India; 53 National Institute of Science Education and Research, HBNI, Bhubaneswar, India; 54 Panjab University, Chandigarh, India; 55 University of Delhi, Delhi, India; 56 Saha Institute of Nuclear Physics, HBNI, Kolkata, India; 57 Indian Institute of Technology Madras, Madras, India; 58 Bhabha Atomic Research Centre, Mumbai, India; 59 Tata Institute of Fundamental Research-A, Mumbai, India; 60 Tata Institute of Fundamental Research-B, Mumbai, India; 61 Indian Institute of Science Education and Research (IISER), Pune, India; 62 Institute for Research in Fundamental Sciences (IPM), Tehran, Iran; 63 University College Dublin, Dublin, Ireland; 64 INFN Sezione di Bari, Università di Bari, Politecnico di Bari, Bari, Italy; 65 INFN Sezione di Bologna, Università di Bologna, Bologna, Italy; 66 INFN Sezione di Catania, Università di Catania, Catania, Italy; 67 INFN Sezione di Firenze, Università di Firenze, Florence, Italy; 68 INFN Laboratori Nazionali di Frascati, Frascati, Italy; 69 INFN Sezione di Genova, Università di Genova, Genova, Italy; 70 INFN Sezione di Milano-Bicocca, Università di Milano-Bicocca, Milan, Italy; 71 INFN Sezione di Napoli, Università di Napoli ’Federico II’, Napoli, Italy, Università della Basilicata, Potenza, Italy, Università G. Marconi, Rom, Italy; 72 INFN Sezione di Padova, Università di Padova, Padova, Italy, Università di Trento, Trento, Italy; 73 INFN Sezione di Pavia, Università di Pavia, Pavia, Italy; 74 INFN Sezione di Perugia, Università di Perugia, Perugia, Italy; 75 INFN Sezione di Pisa, Università di Pisa, Scuola Normale Superiore di Pisa, Pisa, Italy; 76 INFN Sezione di Roma, Sapienza Università di Roma, Rome, Italy; 77 INFN Sezione di Torino, Università di Torino, Torino, Italy, Università del Piemonte Orientale, Novara, Italy; 78 INFN Sezione di Trieste, Università di Trieste, Trieste, Italy; 79 Kyungpook National University, Daegu, South Korea; 80 Chonnam National University, Institute for Universe and Elementary Particles, Kwangju, South Korea; 81 Hanyang University, Seoul, South Korea; 82 Korea University, Seoul, South Korea; 83 Sejong University, Seoul, South Korea; 84 Seoul National University, Seoul, South Korea; 85 University of Seoul, Seoul, South Korea; 86 Sungkyunkwan University, Suwon, South Korea; 87 Vilnius University, Vilnius, Lithuania; 88 National Centre for Particle Physics, Universiti Malaya, Kuala Lumpur, Malaysia; 89 Centro de Investigacion y de Estudios Avanzados del IPN, Mexico City, Mexico; 90 Universidad Iberoamericana, Mexico City, Mexico; 91 Benemerita Universidad Autonoma de Puebla, Puebla, Mexico; 92 Universidad Autónoma de San Luis Potosí, San Luis Potosí, Mexico; 93 University of Auckland, Auckland, New Zealand; 94 University of Canterbury, Christchurch, New Zealand; 95 National Centre for Physics, Quaid-I-Azam University, Islamabad, Pakistan; 96 National Centre for Nuclear Research, Swierk, Poland; 97 Institute of Experimental Physics, Faculty of Physics, University of Warsaw, Warsaw, Poland; 98 Laboratório de Instrumentação e Física Experimental de Partículas, Lisboa, Portugal; 99 Joint Institute for Nuclear Research, Dubna, Russia; 100 Petersburg Nuclear Physics Institute, Gatchina (St. Petersburg), Russia; 101 Institute for Nuclear Research, Moscow, Russia; 102 Institute for Theoretical and Experimental Physics, Moscow, Russia; 103 Moscow Institute of Physics and Technology, Moscow, Russia; 104 National Research Nuclear University ’Moscow Engineering Physics Institute’ (MEPhI), Moscow, Russia; 105 P.N. Lebedev Physical Institute, Moscow, Russia; 106 Skobeltsyn Institute of Nuclear Physics, Lomonosov Moscow State University, Moscow, Russia; 107 Novosibirsk State University (NSU), Novosibirsk, Russia; 108 State Research Center of Russian Federation, Institute for High Energy Physics of NRC “Kurchatov Institute”, Protvino, Russia; 109 National Research Tomsk Polytechnic University, Tomsk, Russia; 110 University of Belgrade, Faculty of Physics and Vinca Institute of Nuclear Sciences, Belgrade, Serbia; 111 Centro de Investigaciones Energéticas Medioambientales y Tecnológicas (CIEMAT), Madrid, Spain; 112 Universidad Autónoma de Madrid, Madrid, Spain; 113 Universidad de Oviedo, Oviedo, Spain; 114 Instituto de Física de Cantabria (IFCA), CSIC-Universidad de Cantabria, Santander, Spain; 115 CERN, European Organization for Nuclear Research, Geneva, Switzerland; 116 Paul Scherrer Institut, Villigen, Switzerland; 117 ETH Zurich-Institute for Particle Physics and Astrophysics (IPA), Zurich, Switzerland; 118 Universität Zürich, Zurich, Switzerland; 119 National Central University, Chung-Li, Taiwan; 120 National Taiwan University (NTU), Taipei, Taiwan; 121 Chulalongkorn University, Faculty of Science, Department of Physics, Bangkok, Thailand; 122 Çukurova University, Physics Department, Science and Art Faculty, Adana, Turkey; 123 Middle East Technical University, Physics Department, Ankara, Turkey; 124 Bogazici University, Istanbul, Turkey; 125 Istanbul Technical University, Istanbul, Turkey; 126 Institute for Scintillation Materials of National Academy of Science of Ukraine, Kharkov, Ukraine; 127 National Scientific Center, Kharkov Institute of Physics and Technology, Kharkov, Ukraine; 128 University of Bristol, Bristol, UK; 129 Rutherford Appleton Laboratory, Didcot, UK; 130 Imperial College, London, UK; 131 Brunel University, Uxbridge, United Kingdom; 132 Baylor University, Waco, USA; 133 Catholic University of America, Washington DC, USA; 134 The University of Alabama, Tuscaloosa, USA; 135 Boston University, Boston, USA; 136 Brown University, Providence, USA; 137 University of California, Davis, Davis, USA; 138 University of California, Los Angeles, USA; 139 University of California, Riverside, Riverside, USA; 140 University of California, San Diego, La Jolla, USA; 141 University of California, Santa Barbara-Department of Physics, Santa Barbara, USA; 142 California Institute of Technology, Pasadena, USA; 143 Carnegie Mellon University, Pittsburgh, USA; 144 University of Colorado Boulder, Boulder, USA; 145 Cornell University, Ithaca, USA; 146 Fermi National Accelerator Laboratory, Batavia, USA; 147 University of Florida, Gainesville, USA; 148 Florida International University, Miami, USA; 149 Florida State University, Tallahassee, USA; 150 Florida Institute of Technology, Melbourne, USA; 151 University of Illinois at Chicago (UIC), Chicago, USA; 152 The University of Iowa, Iowa City, USA; 153 Johns Hopkins University, Baltimore, USA; 154 The University of Kansas, Lawrence, USA; 155 Kansas State University, Manhattan, USA; 156 Lawrence Livermore National Laboratory, Livermore, USA; 157 University of Maryland, College Park, USA; 158 Massachusetts Institute of Technology, Cambridge, USA; 159 University of Minnesota, Minneapolis, USA; 160 University of Mississippi, Oxford, USA; 161 University of Nebraska-Lincoln, Lincoln, USA; 162 State University of New York at Buffalo, Buffalo, USA; 163 Northeastern University, Boston, USA; 164 Northwestern University, Evanston, USA; 165 University of Notre Dame, Notre Dame, USA; 166 The Ohio State University, Columbus, USA; 167 Princeton University, Princeton, USA; 168 University of Puerto Rico, Mayagüez, USA; 169 Purdue University, West Lafayette, USA; 170 Purdue University Northwest, Hammond, USA; 171 Rice University, Houston, USA; 172 University of Rochester, Rochester, USA; 173 Rutgers, The State University of New Jersey, Piscataway, USA; 174 University of Tennessee, Knoxville, USA; 175 Texas A & M University, College Station, USA; 176 Texas Tech University, Lubbock, USA; 177 Vanderbilt University, Nashville, USA; 178 University of Virginia, Charlottesville, USA; 179 Wayne State University, Detroit, USA; 180 University of Wisconsin-Madison, Madison, WI USA; 181 CERN, 1211 Geneva 23, Switzerland

## Abstract

Pseudorapidity, transverse momentum, and multiplicity distributions are measured in the pseudorapidity range $|\eta | < 2.4$ for charged particles with transverse momenta satisfying $p_{\mathrm {T}} > 0.5\,\text {GeV}$ in proton–proton collisions at a center-of-mass energy of $\sqrt{s} = 13\,\text {TeV}$. Measurements are presented in three different event categories. The most inclusive of the categories corresponds to an inelastic $\mathrm {p}$ $\mathrm {p}$ data set, while the other two categories are exclusive subsets of the inelastic sample that are either enhanced or depleted in single diffractive dissociation events. The measurements are compared to predictions from Monte Carlo event generators used to describe high-energy hadronic interactions in collider and cosmic-ray physics.

## Introduction

The study of the properties of particle production without any selection bias arising from requiring the presence of a hard scattering (a selection known as “minimum bias”) is one of the most basic measurements that can be made at hadron colliders. Such events are produced by strong interactions of partons inside the hadrons, which occur at low momentum exchanges, for which predictions of quantum chromodynamics (QCD) cannot be obtained perturbatively, and for which diffractive scatterings or multiple partonic interactions (MPI) play a significant role. The theoretical description of these components of particle production is based on phenomenological models with free parameters adjusted (“tuned”) to reproduce the experimental data. However, when a momentum transfer of several GeV (referred to as a hard process) is involved, predictions obtained from perturbative QCD (pQCD) are, in many cases, in good agreement with the measurements. Understanding the transition region between hard processes calculable with perturbative techniques and soft processes described by nonperturbative models is required for a full description of particle production in proton–proton ($\mathrm {p}$ $\mathrm {p}$) collisions at the LHC. It is also essential when the collider is operated at high luminosities since a bunch crossing contains many $\mathrm {p}$ $\mathrm {p}$ collisions (pileup) forming a complex final state that needs to be theoretically controlled for precise studies of standard model processes, as well as for new physics searches.

Inclusive measurements of charged particle pseudorapidity distributions, $\text {d}{}N_{\text {ch}}/\text {d}{}\eta$, and transverse momentum distributions, $\text {d}{}N_{\text {ch}}/\text {d}{}p_{\mathrm {T}}$, as well as charged particle multiplicities have been previously performed in proton–proton and proton-antiproton collisions in the center-of-mass energy range $\sqrt{s} =$ 0.2–8$\,\text {TeV}$ and in various phase space regions [1–19]. Most of these measurements are described to within 10–20% by present event generators as reported, e.g., in Ref. [20].

More recently, measurements of the charged-hadron pseudorapidity distribution in $\mathrm {p}$ $\mathrm {p}$ collisions at the highest energies reached so far, $\sqrt{s} = 13 \,\text {TeV}$, have been presented in Refs. [21–24]. The present work extends those studies, for charged particles with $p_{\mathrm {T}} > 0.5\,\text {GeV}$ measured over the range $|\eta | < 2.4$, to cover not only the pseudorapidity density, but also the per-event multiplicity probability, $P(N_{\text {ch}})$, as well as different transverse-momentum distributions, such as that of the leading charged particle $\text {d}{}N_{\text {ch}}/\text {d}{}p_{\mathrm {T}}$, and its corresponding integrated spectrum, $D(p_{\text {T,min}})$. The integrated spectrum $D(p_{\text {T,min}})$ is defined as:

1 $$\begin{aligned} D(p_{\text {T,min}}) = \frac{1}{N_{\text {events}}} \int _{p_{\text {T,min}}} \text {d}p_{\text {T,leading}} \left( \frac{\text {d} N}{\text {d} p_{\text {T,leading}}}\right) . \end{aligned}$$

Here $N_{\text {events}}$ is the number of selected events, *N* is the number of events with a leading charged particle with transverse momentum $p_{\text {T,leading}}$, and $p_{\text {T,min}}$ is the lower limit of the integral. In each event, the highest-$p_{\mathrm {T}}$ charged particle within $|\eta | < 2.4$ and with $p_{\mathrm {T}} > 0.5 \,\text {GeV}$ is selected as the leading charged particle. The integrated spectrum of charged particles is sensitive to the transition between the nonperturbative and perturbative QCD regions [17, 25].

The measured distributions are presented for three different event data sets: an inelastic sample, a sample dominated by nonsingle diffractive dissociation events (NSD-enhanced sample), and a sample enriched by single diffractive dissociation events (SD-enhanced sample). The measurements are compared to predictions from different Monte Carlo (MC) event generators used to describe high-energy hadronic interactions in collider and cosmic-ray physics.

This article is organized as follows. Section 2 gives a brief description of the CMS detector. The MC models used for corrections and comparison to data are described in Sect. 3. The data sample, track reconstruction, and event selection are discussed in Sect. 4. The procedure to correct the data for detector effects and the systematic uncertainties affecting the measurements are described in Sects. 5 and 6, respectively. The final results are presented in Sect. 7 and a summary is given in Sect. 8.

## The CMS detector

The central feature of the CMS apparatus is a superconducting solenoid of 6 m internal diameter, providing a magnetic field of 3.8 T. Within the solenoid volume are a silicon pixel and strip tracker, a lead tungstate crystal electromagnetic calorimeter, and a brass and scintillator hadron calorimeter, each composed of a barrel and two endcap sections. Forward calorimeters extend the pseudorapidity coverage provided by the barrel and endcap detectors. Muons are detected in gas-ionization chambers embedded in the steel flux-return yoke outside the solenoid.

The tracking detector consists of 1440 silicon pixel and 15 148 silicon strip detector modules. The barrel is composed of 3 pixel and 10 strip layers around the primary interaction point (IP) at radii ranging from 4.4 to 110 cm. The forward and backward endcaps each consist of 2 pixel disks and 12 strip disks in up to 9 rings. Three of the strip rings and four of the barrel strip layers contain an additional plane, with a stereo angle of 100 mrad, to provide a measurement of the *r*- and *z*-coordinate, respectively. The silicon tracker measures charged particles within the pseudorapidity range $|\eta |< 2.5$. For particles of $1< p_{\mathrm {T}} < 10\,\text {GeV}$ and $|\eta | < 1.4$, the track resolutions are typically 1.5% in $p_{\mathrm {T}}$ and 25–90 (45–150)$\,\mu \text {m}$ in the transverse (longitudinal) impact parameter [26].

The hadron forward (HF) calorimeter uses steel as an absorber and quartz fibers as the sensitive material. The HF calorimeters are located at 11.2 m from the interaction region, one on each end, and together they provide coverage in the range $2.9< |\eta | < 5.2$. Each HF calorimeter consist of 432 readout towers, containing long and short quartz fibers running parallel to the beam. The long fibers run the entire depth of the HF calorimeter (165 cm, or approximately 10 interaction lengths), while the short fibers start at a depth of 22 cm from the front of the detector. By reading out the two sets of fibers separately, it is possible to distinguish showers generated by electrons and photons, which deposit a large fraction of their energy in the long-fiber calorimeter segment, from those generated by hadrons, which produce on average nearly equal signals in both calorimeter segments. Calorimeter towers are formed by grouping bundles of fibers of the same type. Bundles of long fibers form the electromagnetic towers and bundles of short fibers form the hadronic towers.

A more detailed description of the CMS detector, together with a definition of the coordinate system used and the relevant kinematic variables, can be found in Ref. [27].

## Theoretical predictions

Three different event generators simulating hadronic collisions are used to correct the measurements to particle level (Sect. 5), and for comparisons with the final results. Simulated event samples were used to optimize the event selection, vertex selection, and tracking efficiencies.

The pythia 8 (version 8.153) event generator [28] uses a model [28, 29] in which initial-state radiation and multiple partonic interactions are interleaved. Parton showers in pythia are modeled according to the Dokshitzer–Gribov–Lipatov–Altarelli–Parisi (DGLAP) evolution equations [30–32], and hadronization is based on the Lund string fragmentation model [33]. Diffractive cross sections are described by the Schuler–Sjöstrand model [34]. Particle production from a low-mass state X, with $M_\mathrm {X} < 10\,\text {GeV}$, is described by the Lund string fragmentation model, while for higher masses, $M_\mathrm {X} > 10\,\text {GeV}$, a perturbative description of the pomeron–proton scattering is introduced. The latter is based on diffractive parton distribution functions [35–37], which represent probability distributions for partons inside the proton, under the constraint that the proton emerges intact from the collision. The pythia 8 generator is used with the tune cuetp8m1  [20] (also referred to as cuetm1 ), which is based on the Monash tune [38] using the NNPDF2.3LO [39, 40] parton distribution function (PDF) set, with parameters optimized to reproduce underlying event (UE) data from CMS at $\sqrt{s} = 7 \,\text {TeV}$ and CDF at $\sqrt{s} = 1.96 \,\text {TeV}$.

The minimum bias Rockefeller (MBR) model [41] is also implemented within the pythia 8 event generator. When used in conjunction with the 4c tune [42] (which includes parton showering) it is referred to as the pythia 8 mbr4c model. This model reproduces the measured energy dependence of the total, elastic, and inelastic $\mathrm {p}$ $\mathrm {p}$ cross sections, and can be used to fully simulate the main diffractive components of the inelastic cross section. The generation of diffractive processes is based on a phenomenological renormalized Regge model [43, 44], interpreting the pomeron flux as the probability of forming a diffractive rapidity gap. The value of the pomeron intercept $\alpha (0)=1.08$ is found to give the best description of the diffractive dissociation cross sections measured by CMS at $\sqrt{s}=7\,\text {TeV}$ [45].

The data are also compared to predictions from the epos  [46] MC event generator (version 1.99) used in cosmic ray physics [47], including contributions from soft- and hard-parton dynamics. The soft component is described in terms of the exchange of virtual quasi-particle states, as in Gribov’s Reggeon field theory [48], with multi-pomeron exchanges accounting for UE effects. At higher energies, the interaction is described in terms of the same degrees of freedom (reggeons and pomerons), but generalized to include hard processes via hard-pomeron scattering diagrams, which are equivalent to a leading order pQCD approach with DGLAP evolution. The epos generator is used with the LHC tune [49, 50].

Event samples obtained from the event generators pythia 8, pythia 8 mbr, and epos are passed through the CMS detector simulation based on geant4  [51], and are processed and reconstructed in the same manner as collision data. The number of pileup interactions in the MC samples is adjusted to match the distribution in the data.

## Data set, track reconstruction, and event selection

In order to minimize the effect of pileup, the data considered in the analysis were collected in a special run in summer 2015 with an average number of $\mathrm {p}$ $\mathrm {p}$ interactions per bunch crossing of 1.3 [52].

The two LHC beam position monitors closest to the IP for each LHC experiment, called the beam pick-up timing experiment (BPTX) detectors, are used to trigger the detector readout. They are located around the beam pipe at a distance of 175 m from the IP on either side, and are designed to provide precise information on the bunch structure and timing of the incoming beams. Events are selected by requiring the presence of both beams crossing at the IP, as inferred from the BPTX detectors.

The CMS track reconstruction algorithm is based on a combinatorial track finder (CTF) [53]. The collection of reconstructed tracks is obtained through multiple iterations of the CTF reconstruction sequence. The iterative tracking sequence consists of six iterations. The first iteration is designed to reconstruct prompt tracks (originating near the $\mathrm {p}$ $\mathrm {p}$ interaction point) with three pixel hits and $p_{\mathrm {T}} > 0.8\,\text {GeV}$. The subsequent iterations are intended to recover prompt tracks that only have two pixel hits or lower $p_{\mathrm {T}}$. At each iteration an extrapolation of the trajectory is performed, and using the Kalman filter, additional strip hits compatible with the trajectory are assigned.

High-purity tracks [26] are selected with a reconstructed $p_{\mathrm {T}} > 0.5\,\text {GeV}$, in order to have high tracking efficiency (${>}80\%$) and a relative transverse momentum uncertainty smaller than 10%. Tracks are measured within the pseudorapidity range $|\eta | < 2.4$ corresponding to the fiducial acceptance of the tracker, in order to avoid effects from tracks very close to its geometric edge at $|\eta |=2.5$. The impact parameter with respect to the beam spot in the transverse plane, $d_{xy}$, is required to satisfy $|d_{xy}/\sigma _{xy} | < 3$, while for the point of closest approach to the primary vertex along the *z*-direction, ($d_{z}$), the requirement $|d_z/\sigma _z | < 3$ is imposed. Here $\sigma _{xy}$ and $\sigma _z$ denote the uncertainties in $d_{xy}$ and $d_{z}$, respectively. The number of pixel detector hits associated with a track has to be at least 3 in the pseudorapidity region $|\eta | \le 1$ and at least 2 for $|\eta | > 1$.

Rejection of beam background events and events with more than one collision per bunch crossing is achieved by requiring exactly one reconstructed primary vertex [26]. The vertex produced by each collision is required to be within $|z | < 15\,\text {cm}$ with respect to the center of the luminous region along the beamline and within $0.2\,\text {cm}$ in the transverse direction.

Different event classes are defined based on activity in the HF calorimeters by requiring the presence of at least one tower with an energy above the threshold value of 5$\,\text {GeV}$ in the fiducial acceptance region, $3< |\eta | < 5$. The veto condition is defined by an energy deposit in the towers less than a given threshold value. An inelastic sample consists of events with activity on at least one side of the calorimeters, whereas an NSD-enhanced sample contains those with calorimeter activity on both sides. An SD-enhanced sample is defined by requiring activity on only one side of the calorimeters, with a veto condition being applied to the other side.

The HF energy threshold of 5$\,\text {GeV}$ was determined from the measurement of electronic noise and beam-induced background in the HF calorimeters, using an event sample for which a single beam was circulating in the LHC ring, and an event sample without beams. The threshold of 5$\,\text {GeV}$ keeps the background due to noise low, while still maintaining a high selection efficiency. The fraction of events with at least one HF tower on either side of the detector with an energy above the threshold of 5$\,\text {GeV}$ in the event samples with no collisions (one beam or no beam) is 0.13%. The efficiency of the event selection defined by the presence of at least one tower with an energy above 5$\,\text {GeV}$ in either side of the calorimeters is 99.3%, and is calculated with respect to the event sample defined by the presence of exactly one reconstructed primary vertex.

In total a sample of 2.23 million events is selected containing 2 million NSD events and 0.23 million SD-enhanced events.

## Correction to particle level

The data are corrected for tracking and event selection efficiencies, as well as for detector resolution effects. The corrected distributions correspond to stable primary charged particles, which are either directly produced in $\mathrm {p}$ $\mathrm {p}$ collisions or result from decays of particles with decay length < 1 cm. At particle level, events are selected if at least one charged particle is found within $|\eta | < 2.4$ and $p_{\mathrm {T}} > 0.5\,\text {GeV}$. The different event selections are defined in a similar manner to how they are defined at detector level in order to avoid any bias towards a specific MC model. Activity in the forward region is defined by the presence of at least one particle, either charged or neutral, with an energy above 5$\,\text {GeV}$ in the region $3< |\eta | < 5$ (referred to as the trigger particle). The veto condition is equivalently defined by the absence of particles with an energy above 5$\,\text {GeV}$. The inelastic data set is defined by requiring a trigger particle in the pseudorapidity range $3< \eta < 5$ or $-5< \eta < -3$. The NSD-enhanced event sample is defined by requiring a trigger particle in the regions $3< \eta < 5$ and $-5< \eta < -3$. The SD-enhanced event sample is defined by requiring a trigger particle in either the positive or negative $\eta$ range, with the veto condition applied to the other region. The SD-enhanced event sample is further divided into two exclusive subsets according to the $\eta$ region in which the trigger particle is detected. These subsets are referred to as SD-One-Side enhanced event samples. Table 1 shows a summary of the event selection definitions at particle level.

**Table 1:** Summary of stable-particle level definitions for each of the event samples, corresponding to the inelastic, the NSD-enhanced, and SD-enhanced categories. Charged particles are selected with $p_{\mathrm {T}} > 0.5\,\text {GeV}$ and $|\eta | < 2.4$. Forward trigger particles correspond to those with energy $E> 5\,\text {GeV}$ located in side$^-$ (defined as $-5< \eta < -3$) and/or side$^+$ (defined as $3< \eta < 5$). Similarly, a veto corresponds to the absence of a trigger particle with $E> 5\,\text {GeV}$ in side$^-$ and/or side$^+$

Event sample	Forward region energy selection
Inelastic	Trigger particle in side$^-$ or side$^+$
NSD-enhanced	Trigger particle in side$^-$ and side$^+$
SD-enhanced	Trigger particle in side$^-$( side$^+$) and veto in side$^+$( side$^-$)

A response matrix, *R*, is constructed using the information provided by the MC event generators and by the full detector simulation. The elements of the response matrix ($R_{ji}$) represent the conditional probability that (for a given observable) a true value *i* is measured as a value *j*. In this analysis, two different correction procedures were implemented. The first method (method 1) makes use of the full detector simulation, while in the second method (method 2) a parametrization of the detector response is implemented in order to overcome the statistical limitations of the full detector simulation. Whenever it is possible to accurately parametrize the detector resolution, method 2 is used, otherwise method 1 is applied. The two implemented methods account for unreconstructed particles (misses) and wrongly reconstructed tracks (misreconstruction), as well as for the event selection efficiency including the vertex and enhanced-event selection. In method 1, the correction is performed in two steps. The first step corrects for detector resolution, missed particles and misreconstructed tracks, using an unfolding procedure. The second step corrects for the event selection efficiency. In method 2, the *R* matrix is constructed in a manner that does not include information on the missed particles and misreconstructed tracks; therefore a correction factor to account for these effects is applied. This correction factor takes into account the missed particles and the misreconstructed tracks, as well as the event selection efficiency. In contrast to method 1, this correction factor is applied as a function of the observable of interest. The reason for this is that the number of missing particles in the reconstruction and the number of misreconstructed tracks both show a dependence on the different observables. The D’Agostini method [54] is used to unfold the detector effects.

The final corrected distributions are obtained by taking the average of two corrected distributions, each corrected using one of the two MC models that describe best the data at detector level. Most of the distributions are best described by pythia 8 cuetm1 and epos LHC. The only exception is the pseudorapidity distribution of the SD-enhanced event sample, for which pythia 8 cuetm1 and pythia 8 mbr4c have been used.

## Systematic uncertainties

The following sources of systematic uncertainty are taken into account: tracking efficiency, description of the pileup modeling, sensitivity to the specific value used for the energy threshold applied to the HF towers, and the dependence on the model used for the corrections.

The systematic uncertainties show almost no dependence on the pseudorapidity of the particles in the fiducial region considered here. For the charged particle multiplicity and $p_{\mathrm {T}}$ distributions, the systematic uncertainties are dependent on the value of the measured observable. The systematic uncertainties associated with the $p_{\mathrm {T}}$ distributions of all charged particles, the leading charged particle, and the integrated spectrum of the latter show a similar behavior.

- Tracking efficiency. The systematic uncertainty due to the difference between the track reconstruction efficiency in data and simulation is $\approx 4$%. This has been obtained in Ref. [16] at $\sqrt{s}=8\,\text {TeV}$ and validated for $\sqrt{s}=13\,\text {TeV}$ data by comparing the tracking performance of the data set used in this analysis to the performance of the same data set but reconstructed with the (different) tracking conditions used in Ref. [16]. The tracking efficiency is estimated with a data-driven method known as “tag-and-probe” [55] by exploiting resonances decaying into two particles.
- Pileup modeling. The systematic uncertainty associated with the modeling of the pileup contribution is calculated by varying the nominal selection of events with exactly one vertex to that obtained with at least one vertex where only the tracks associated with the vertex with the largest sum of the squared transverse momenta of its tracks are used. The difference between these two selections is taken as the associated uncertainty. For the pseudorapidity distributions, the uncertainty is estimated to be about 1% for the inelastic event sample, while it is about 1.5 and 0.3% for the NSD- and SD-enhanced event samples, respectively. An uncertainty of about 2% on the $p_{\mathrm {T}}$ distributions for the most inclusive selection procedure is obtained, while it is smaller than 1% for the SD-enhanced event sample. The inelastic and NSD-enhanced event samples have similar uncertainties for the multiplicity distributions, first increasing at low multiplicities from 2 to 8% and then decreasing to 0.5% for large multiplicities.
- Event selection with HF. The systematic uncertainty associated with the event selection is determined by varying the threshold applied to the energy of the HF calorimeter towers, while keeping the definition of the stable-particle level unchanged. The default value of the energy threshold applied to the HF calorimeter towers is varied from 5$\,\text {GeV}$ by $\pm 1 \,\text {GeV}$.
  In the inelastic and NSD-enhanced event samples an uncertainty of less than 2% for all the relevant distributions is obtained, while for the SD-enhanced sample the uncertainty increases to $\approx 6$% for the pseudorapidity distributions and varies between 1 and 15% for the $p_{\mathrm {T}}$ distributions.
- Model dependence. The systematic uncertainty due to the model dependence is calculated as one half of the difference between the corrected distributions using the two MC models mentioned in Sect. 5. For the pseudorapidity distributions, it varies between 0.1 and 1% for the inelastic and NSD-enhanced event samples, and is about 7% for the SD-enhanced sample.
  For the transverse-momentum distributions, the most inclusive event samples have a maximum uncertainty of about 4% at high $p_{\mathrm {T}}$, while the SD-enhanced event sample exhibits a maximum uncertainty of 10% around 2$\,\text {GeV}$, decreasing at both the low and high ends of the spectrum.
  For the event multiplicity distributions, the inelastic and NSD-enhanced event samples have similar uncertainties with values up to 8%, reaching a maximum uncertainty for low and high multiplicities, and a minimum for multiplicities between 5 and 40.

The total systematic uncertainty is obtained by adding the different sources discussed above in quadrature. Table 2 summarizes all the contributions per observable and per event selection. The total uncertainty is reported for each case.

Due to the statistical limitations of the multiplicity measurement in the SD-enhanced event sample, this distribution is not included in the results.

**Table 2:** Summary of systematic uncertainties per observable for each of the event samples. The observables are (presented as rows, from top to bottom) pseudorapidity, multiplicity, transverse momentum, leading transverse momentum, and the integral of the latter. The columns, from left to right, represent the following event samples: Inelastic, NSD-enhanced and SD-enhanced. For each observable the respective sources of uncertainty are listed. These are, from top to bottom: the tracking efficiency, the pileup modelling, the event selection and the model dependence. The final value in each case represents the total systematic uncertainty

Systematic uncertainties (%)				
Observable	Source	Inelastic	NSD-enhanced	SD-enhanced
$\frac{\text {d}N_{\text {ch}}}{\text {d}\eta }$	Tracking efficiency	4	4	4
	Pileup modeling	1	1.6	0.3
	Event selection	$<0.2$	1	7
	Model dependence	0.8	0.5	7
	Total	4	4	9
$P(N_{\text {ch}})$	Tracking efficiency	4	4	—
	Pileup modeling	0.5–8	0.5–8	—
	Event selection	0–2	0–2	—
	Model dependence	0–8	0–8	—
	Total	4–8	4–8	—
$\frac{\text {d}N_{\text {ch}}}{\text {d}p_{\mathrm {T}}}$	Tracking efficiency	4	4	4
	Pileup modeling	0–2	0.5–2	0–2
	Event selection	$<0.2$	1	1–12
	Model dependence	0–4	0–4	4–10
	Total	4	4	7 – 14
$\frac{\text {d}N_{\text {ch}}}{\text {d}{p_{\text {T,leading}}}}$	Tracking efficiency	4	4	4
	Pileup modeling	0–4	0.5–4	0–4
	Event selection	$<0.2$	1	1–12
	Model dependence	0–3	0–3	4–14
	Total	4	4	5–14
$D(p_{\text {T,min}})$	Tracking efficiency	4	4	4
	Pileup modeling	0–2	0.5–2	0–2
	Event selection	$<0.2$	1	1–12
	Model dependence	0–4	0–4	1–11
	Total	4	4	4–15

## Results

**Fig. 1:**
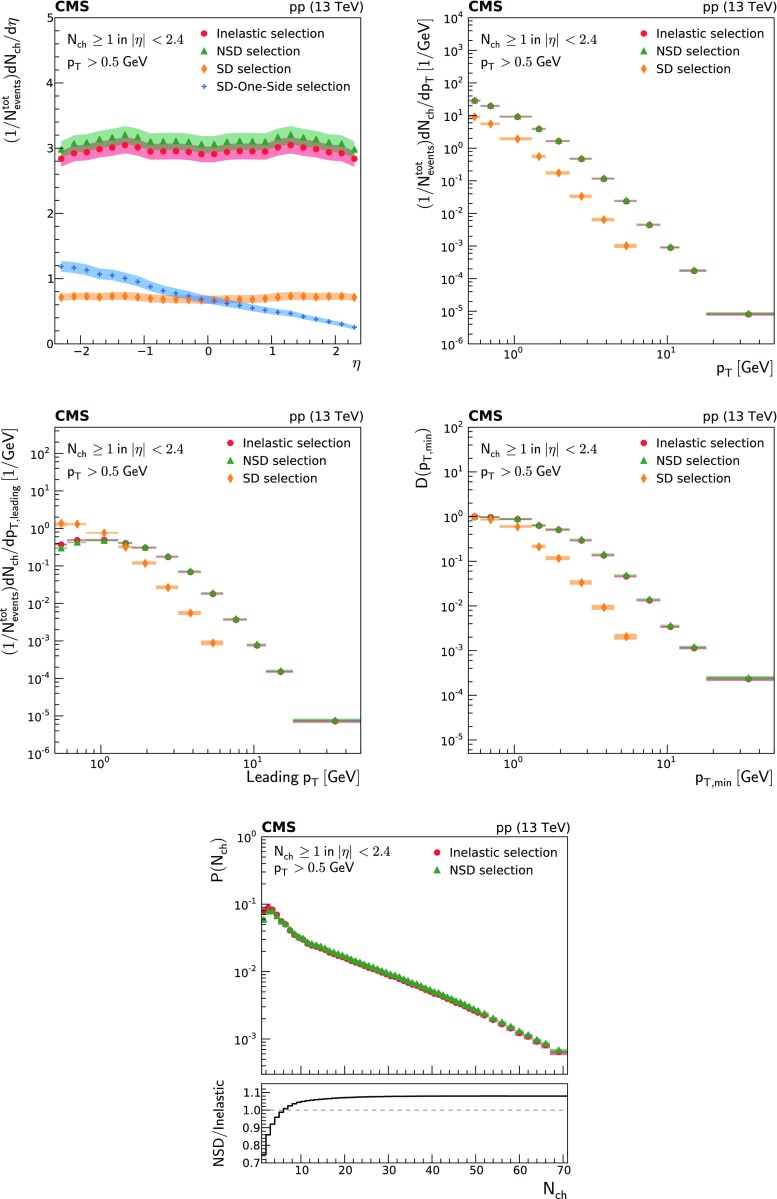
From top to bottom, left to right: pseudorapidity , $p_{\mathrm {T}}$, leading $p_{\mathrm {T}}$, integrated leading $p_{\mathrm {T}}$, and multiplicity of charged particles per event for the inelastic (circles), NSD-enhanced (triangles), SD-enhanced (diamonds), and SD-One-Side enhanced (crosses) event samples. The band encompassing the data points represent the total systematic uncertainty, while the statistical uncertainty is included as a vertical bar for each data point

Charged particle distributions corrected to particle level as a function of $\eta$, $p_{\mathrm {T}}$ and leading $p_{\mathrm {T}}$, as well as the integrated leading $p_{\mathrm {T}}$ as a function of $p_{\text {T,min}}$ ($D(p_{\text {T,min}})$), and the multiplicity per event $P(N_{\text {ch}})$ are shown in Fig. 1. They are presented for the different event categories corresponding to the most inclusive (inelastic), the diffraction-depleted (NSD), and the diffraction-enhanced (SD) samples.

The SD-minus and SD-plus samples are mutually exclusive, depending on the side of the forward-detector that contains the hadronic activity. The pseudorapidity distribution of the SD-enhanced event sample is also presented as a symmetrized distribution constructed from the SD-minus and SD-plus enhanced samples and is referred to as the SD-One-Side enhanced event sample. The symmetrization is performed by reflecting the distribution with respect to $\eta = 0$. The pseudorapidity distributions are averaged over the positive and negative $\eta$ ranges to suppress statistical fluctuations.

The per-event yields, defined in Eq. (1), are obtained experimentally as

2 $$\begin{aligned} {D}(p_{\text {T,min}}) = \frac{1}{N_{\text {events}}} \sum _{p_{\text {T,leading}} >p_{\text {T,min}}} \varDelta p_{\text {T,leading}} \left( \frac{\text {d} \varDelta N}{\text {d} \varDelta p_{\text {T,leading}}}\right) , \end{aligned}$$

where $N_{\text {events}}$ is the number of events with a leading charged particle, $\varDelta p_{\text {T,leading}}$ is the bin width, and $\varDelta N$ is the number of events with a leading charged particle in each bin.

In general terms, the inelastic and NSD distributions are similar. The pseudorapidity density of the SD-enhanced event sample is about a factor of 4 lower than that of the most inclusive event samples. The $p_{\mathrm {T}}$ distributions (i.e., $p_{\mathrm {T}}$, leading $p_{\mathrm {T}}$, and integrated leading $p_{\mathrm {T}}$) of the SD-enhanced event sample fall very steeply for large $p_{\mathrm {T}}$ values. The charged particle multiplicity distribution of the NSD-enhanced event sample shows a depletion of low-multiplicity events and an increase of high-multiplicity events compared to that of the inelastic sample.

**Fig. 2:**
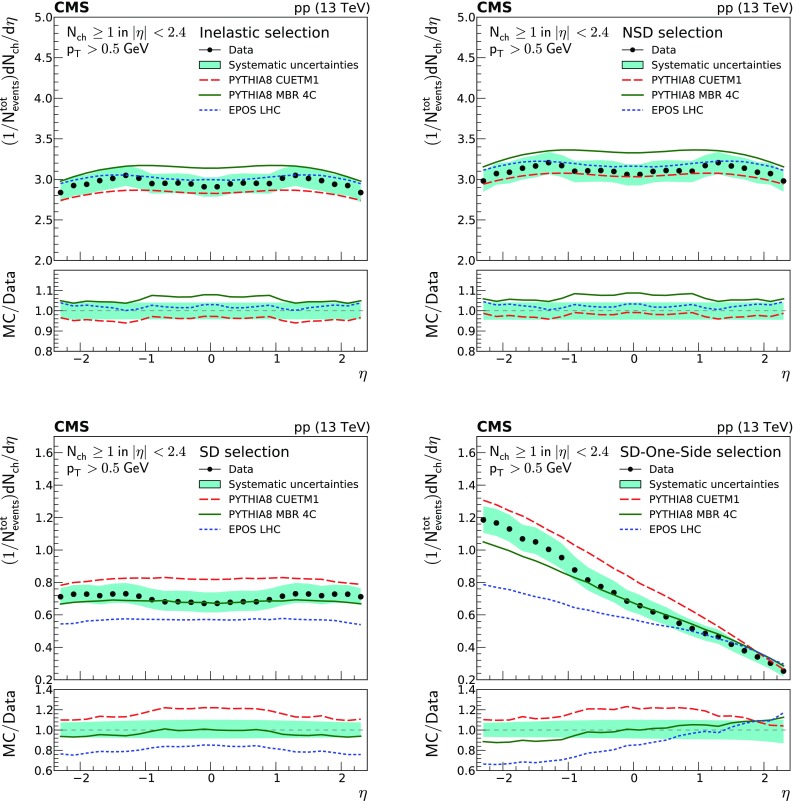
Charged particle pseudorapidity densities averaged over both positive and negative $\eta$ ranges. Top to bottom, left to right: inelastic, NSD-, SD-, and SD-One-Side enhanced event samples. The measurements are compared to the predictions of the pythia 8 cuetm1 (long dashes), pythia 8 mbr4c (continuous line), and epos LHC (short dashes) event generators. The band encompassing the data points represent the total systematic uncertainty, while the statistical uncertainty is included as a vertical bar for each data point. The lower panels show the corresponding MC-to-data ratios

Figure 2 shows the pseudorapidity densities of charged particles for four different event categories. The measurements are compared to the predictions of different MC event generators, namely pythia 8 cuetm1, pythia 8 mbr4c, and epos LHC. The predictions of epos LHC provide the best description of the data within uncertainties for the inelastic event sample. The predictions of pythia 8 cuetm1 slightly underestimate the measurements, while those of pythia 8 mbr4c overestimate them. For the NSD-enhanced event sample, the predictions of epos LHC and pythia 8 cuetm1 both give a reasonable description of the data within uncertainties, while the predictions of pythia 8 mbr4c overestimate the measurements. The opposite behavior is observed for the SD-enhanced event samples, with the predictions of epos LHC underestimating the data, and the predictions of pythia 8 cuetm1 overestimating them. The predictions from pythia 8 mbr4c describe well the SD data within uncertainties, showing only small deviations at the edges of the phase space. The SD-One-Side enhanced event sample is not well described by epos LHC, while pythia 8 cuetm1 tends to overestimate data, and the prediction from pythia 8 mbr4c describes the measurements within uncertainties over almost the full range, exhibiting some deviations in the regions where the diffractive dissociative system is observed.

**Fig. 3:**
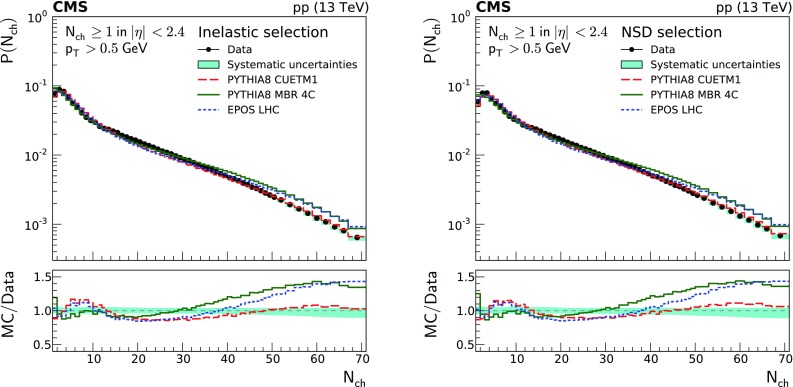
Charged particle multiplicity distributions of the inelastic (left), and NSD-enhanced (right) event samples. The measurements are compared to the predictions of the pythia 8 cuetm1 (long dashes), pythia 8 mbr4c (continuous line), and epos LHC (short dashes) event generators. The band encompassing the data points represent the total systematic uncertainty, while the statistical uncertainty is included as a vertical bar for each data point. The lower panels show the corresponding MC-to-data ratios

Figure 3 shows the charged particle multiplicity distributions for the inelastic and NSD-enhanced event samples. The different event generators provide similar predictions for the inelastic and NSD-enhanced event samples, with differences appearing only at low multiplicities. It is in this low-multiplicity regime that the SD dissociation events contribute the most. The pythia 8 mbr4c generator gives the best description of the data in the low-multiplicity region, while pythia 8 cuetm1 and epos LHC overestimate the data by approximately 20%. This behavior is similar to that observed in the pseudorapidity distribution of the SD-enhanced selection, where pythia 8 mbr4c provides the best description of the SD-enhanced event sample. For multiplicities above 35, the predictions of pythia 8 cuetm1 give the best description of the data, whereas those of pythia 8 mbr4c and epos LHC are off by up to 50%. The high multiplicity region is especially sensitive to MPI and improving its modelling could lead to a better understanding of these processes.

**Fig. 4:**
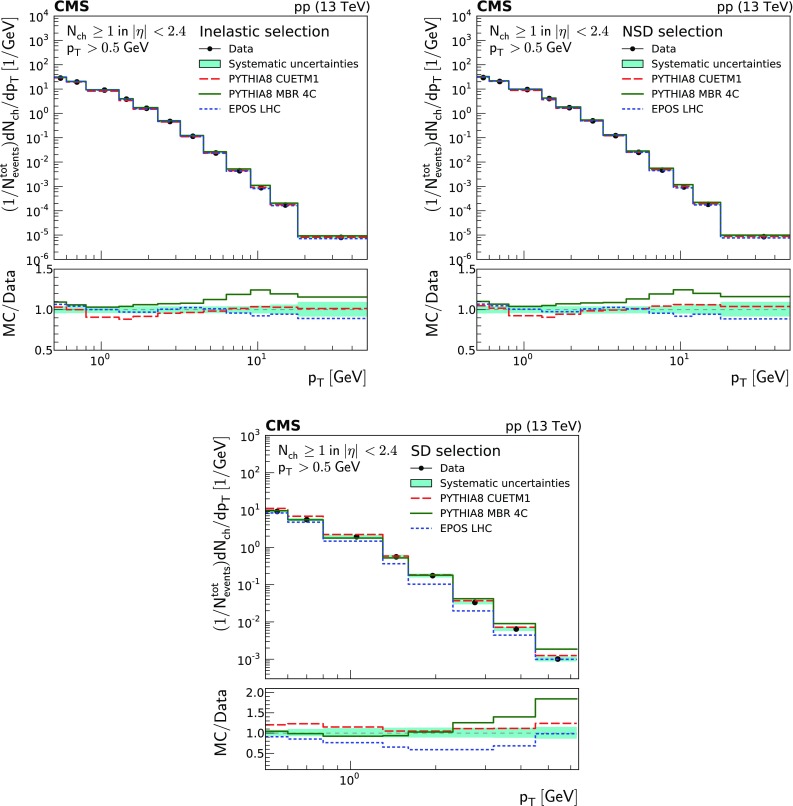
Charged particle transverse-momentum densities of inelastic (top left), NSD-enhanced (top right), and SD-enhanced (bottom) event samples. The measurements are compared to the predictions of the pythia 8 cuetm1 (long dashes), pythia 8 mbr4c (continuous line), and epos LHC (short dashes) event generators. The band encompassing the data points represent the total systematic uncertainty, while the statistical uncertainty is included as a vertical bar for each data point. The lower panels show the corresponding MC-to-data ratios

**Fig. 5:**
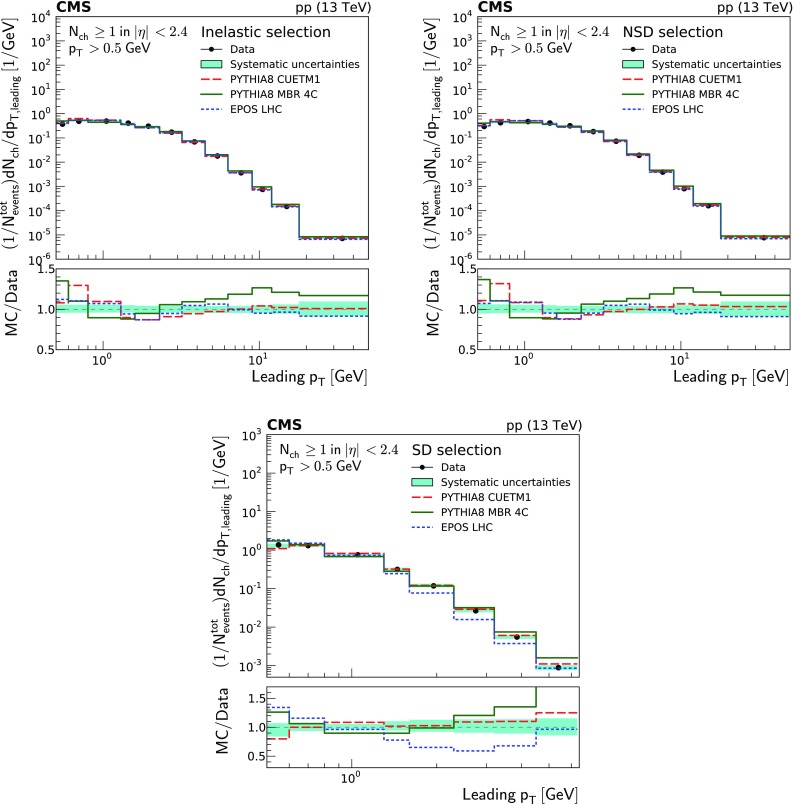
Leading charged particle $p_{\mathrm {T}}$ distributions of inelastic (top left), NSD-enhanced (top right), and SD-enhanced (bottom) event samples. The measurements are compared to the predictions of the pythia 8 cuetm1 (long dashes), pythia 8 mbr4c (continuous line), and epos LHC (short dashes) event generators. The band encompassing the data points represent the total systematic uncertainty, while the statistical uncertainty is included as a vertical bar for each data point. The lower panels show the corresponding MC-to-data ratios

**Fig. 6:**
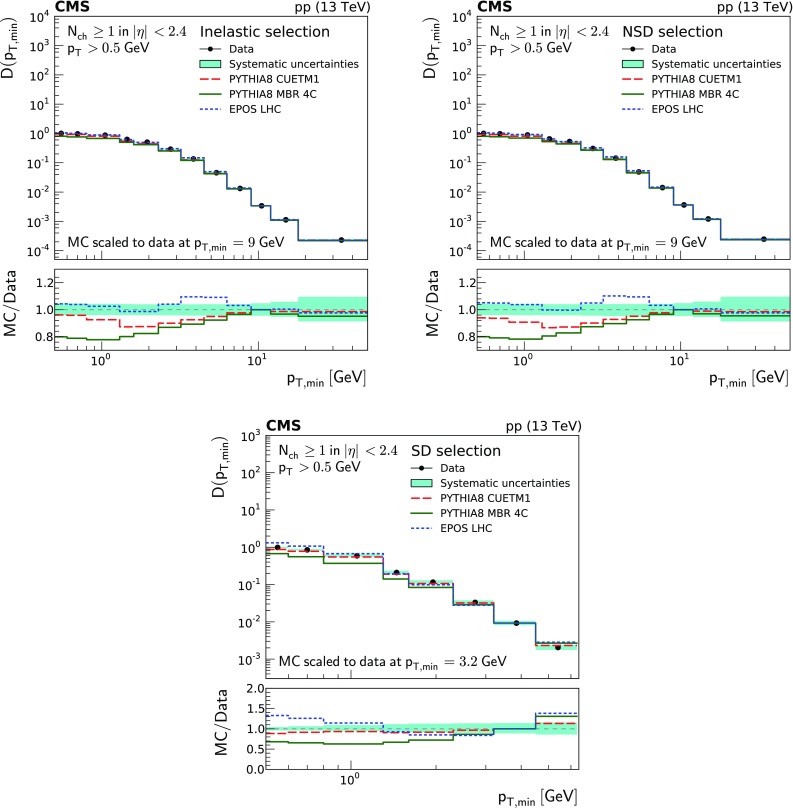
Integrated leading charged particle $p_{\mathrm {T}}$ distributions as a function of $p_{\text {T,min}}$ for inelastic (top left), NSD-enhanced (top right), and SD-enhanced (bottom) event samples. The measurements are compared to the predictions of the pythia 8 cuetm1 (long dashes), pythia 8 mbr4c (continuous line), and epos LHC (short dashes) event generators. The band encompassing the data points represent the total systematic uncertainty, while the statistical uncertainty is included as a vertical bar for each data point. The lower panels show the corresponding MC-to-data ratios

Figures 4, 5 and 6 show the charged particle $p_{\mathrm {T}}$ distributions for all the particles, the leading particle, and the integrated spectrum of the latter, for the inelastic, NSD-, and SD-enhanced event samples. The $p_{\mathrm {T}}$ range for the SD-enhanced event sample is smaller compared to the other samples, ranging up to 6.3$\,\text {GeV}$ instead of 50$\,\text {GeV}$. This is a consequence of the more steeply falling $p_{\mathrm {T}}$ spectrum of the SD-enhanced event sample with respect to the other two event categories (Fig. 1).

The $p_{\mathrm {T}}$ distributions of the charged particles in the inelastic and NSD-enhanced event samples are best described by the predictions of pythia 8 cuetm1 over almost the full $p_{\mathrm {T}}$ range. Small deviations of up to 10% in the low-$p_{\mathrm {T}}$ region are observed. This region is dominated by particles coming from MPI. The predictions of pythia 8 mbr4c describe the low-$p_{\mathrm {T}}$ region but rapidly start to overestimate particle production for $p_{\mathrm {T}} >5\,\text {GeV}$ by up to 30%. The predictions of epos LHC give a reasonable description of the data for transverse momenta up to $p_{\mathrm {T}} \approx 10$ $\,\text {GeV}$, while above this value they underestimate it by $\approx 10$%. In the case of the SD-enhanced event sample, pythia 8 cuetm1 gives the best description of the data, while epos LHC and pythia 8 mbr4c underestimate or overestimate the data by 40 and 80%, respectively. It is interesting to observe how difficult it is to simultaneously describe within a given model both the bulk of soft particles mainly coming from MPI and the high-$p_{\mathrm {T}}$ particles primarily coming from hard parton scattering.

The leading $p_{\mathrm {T}}$ distributions of charged particles and their integral as a function of $p_{\text {T,min}}$ are presented in Figs. 5 and 6, respectively. These two distributions provide valuable information on the modeling of the transition between the nonperturbative and perturbative regimes, and on the modeling of MPI [25]. For the case of the leading transverse momentum distributions, the predictions of epos LHC give the best description of the data for the inelastic and NSD-enhanced event samples almost everywhere within the experimental uncertainties with only some small deviations at low-$p_{\mathrm {T}}$ values of up to $\approx 10$%. For $p_{\mathrm {T}} > 4 \,\text {GeV}$, the predictions of pythia 8 cuetm1 are able to reproduce these data. The predictions of pythia 8 mbr4c are not able to describe the data at either low or high $p_{\mathrm {T}}$ for any of the analyzed event samples. In the case of the SD-enhanced event sample, the predictions of pythia 8 cuetm1 provide the best description of the data, while those of epos LHC disagree by up to $\approx 40$%.

For the distribution of the integrated leading charged particle $p_{\mathrm {T}}$ as a function of $p_{\text {T,min}}$, the predictions are normalized to the data in the high-$p_{\text {T,min}}$ region, since this region is better described by the models. The $p_{\text {T,min}}$ distribution for the SD-enhanced event sample is very different from the others, and the normalization is performed at $p_{\text {T,min}} =3.2\,\text {GeV}$, while for inelastic and NSD-enhanced samples it is performed at $p_{\text {T,min}} = 9 \,\text {GeV}$. The inelastic and NSD-enhanced event samples are best described by the predictions of epos LHC and pythia 8 cuetm1 , although the former overestimates particle production by about $10\%$ at around 4–5$\,\text {GeV}$, and the latter underestimates it by a similar amount at around $p_{\text {T,min}} =1\,\text {GeV}$. The predictions of pythia 8 mbr4c agree with the data in the high-$p_{\mathrm {T}}$ region above 9$\,\text {GeV}$ but increasingly underestimate the data at lower $p_{\mathrm {T}}$ values, where discrepancies of up to about $20\%$ are observed. The predictions of pythia 8 cuetm1 describe best the SD-enhanced data set, while those of epos LHC and pythia 8 mbr4c overestimate and underestimate the data by up to about $40\%$, respectively. Comparing the shapes of the $D(p_{\text {T,min}})$ distributions for the inelastic (or NSD-enhanced) and SD-enhanced samples, the transition between the regions dominated by particle production from MPIs (and softer diffractive scatterings) and from single-hard parton scatterings seemingly occurs at about 4 and $2 \,\text {GeV}$, respectively, as indicated by the (fast) change of slope in the spectra around these $p_{\text {T,min}}$ values.

## Summary

Charged particle distributions measured with the CMS detector in minimum bias proton–proton collisions at a center-of-mass energy of $\sqrt{s}=13\,\text {TeV}$ have been presented. Charged particles are selected with transverse momenta satisfying $p_{\mathrm {T}} >0.5\,\text {GeV}$ in the pseudorapidity range $|\eta | < 2.4$. The measured distributions, corrected for detector effects, are presented for three different event samples selected according to the maximum particle energy in the range $3< |\eta | < 5$. The event samples correspond to an inelastic sample, a sample dominated by nonsingle diffractive dissociation events (NSD-enhanced sample), and an event sample enriched by single diffractive dissociation events (SD-enhanced sample).

In general, the event generators epos LHC , pythia 8 cuetm1, and pythia 8 mbr4c describe the measurements reasonably well. However, differences are observed in the pseudorapidity distributions for the SD-enhanced event sample in the region where the diffractive dissociative system is observed. In the distributions integrated over the $p_{\mathrm {T}}$ of the leading particle above a given threshold, $D(p_{\text {T,min}})$, deviations of up to 40% are observed in the small $p_{\mathrm {T}}$ region. The change from a relatively flat to a falling $D(p_{\text {T,min}})$ distribution occurs at different $p_{\text {T,min}}$ values for the diffractive-enhanced event samples ($p_{\text {T,min}} \approx 2$ $\,\text {GeV}$) and the inelastic and NSD-enhanced sample ($p_{\text {T,min}} \approx 4$ $\,\text {GeV}$).

The level of agreement between these new measurements at $\sqrt{s}=13\,\text {TeV}$ and the event generators predictions is comparable to that observed for previous measurements at lower energies. The measurements described here provide new insights into low momentum-exchange parton scatterings that dominate inelastic (including diffractive) $\mathrm {p}$ $\mathrm {p}$ interactions. The rich variety of distributions presented for different event samples, especially those enhanced in diffractive processes, provide new information to understand the transition from perturbative to nonperturbative regions in particle production in high-energy $\mathrm {p}$ $\mathrm {p}$ collisions and help constrain model parameters in modern hadronic event generators used in collider and cosmic-ray physics.
